# High level expression and facile purification of recombinant silk-elastin-like polymers in auto induction shake flask cultures

**DOI:** 10.1186/2191-0855-3-11

**Published:** 2013-02-05

**Authors:** Raul Machado, João Azevedo-Silva, Cristina Correia, Tony Collins, Francisco Javier Arias, Jose Carlos Rodríguez-Cabello, Margarida Casal

**Affiliations:** 1 CBMA (Centre of Molecular and Environmental Biology), Department of Biology, University of Minho, Campus de Gualtar, 4710-057, Braga, Portugal; 2 Bioforge (Group for Advanced Materials and Nanobiotechnology), Centro I+D, Universidad de Valladolid, Valladolid, Spain; 3 Networking Research Centre on Bioengineering, Biomaterials and Nanomedicine (CIBER-BBN), 47011, Valladolid, Spain

**Keywords:** Silk-elastin-like polymers, Recombinant protein expression, Auto induction, Acid-based cell lysis, Ammonium sulphate purification, Non-chromatographic purification

## Abstract

Silk-elastin-like polymers (SELPs) are protein-based polymers composed of repetitive amino acid sequence motifs found in silk fibroin (GAGAGS) and mammalian elastin (VPGVG). These polymers are of much interest, both from a fundamental and applied point of view, finding potential application in biomedicine, nanotechnology and as materials. The successful employment of such polymers in such diverse fields, however, requires the ready availability of a variety of different forms with novel enhanced properties and which can be simply prepared in large quantities on an industrial scale. In an attempt to create new polymer designs with improved properties and applicability, we have developed four novel SELPs wherein the elastomer forming sequence poly(VPGVG) is replaced with a plastic-like forming sequence, poly(VPAVG), and combined in varying proportions with the silk motif. Furthermore, we optimised a simplified production procedure for these, making use of an autoinduction medium to reduce process intervention and with the production level obtained being 6-fold higher than previously reported for other SELPs, with volumetric productivities above 150 mg/L. Finally, we took advantage of the known enhanced stability of these polymers in developing an abridged, non-chromatographic downstream processing and purification protocol. A simple acid treatment allowed for cell disruption and the obtention of relative pure SELP in one-step, with ammonium sulphate precipitation being subsequently used to enable improved purity. These simplified production and purification procedures improve process efficiency and reduce costs in the preparation of these novel polymers and enhances their potential for application.

## Introduction

Nature has refined structural proteins to perform defined functions through the association of specific amino acid sequences in various combinations, creating diverse materials with remarkable properties. These have served as a source of inspiration for a new class of bio-engineered materials, the protein-based polymers (PBPs), founded on conservative amino acid motifs found in nature. These bioinspired materials exhibit the properties of natural proteins but frequently also display functions that are not found in nature (Rodriguez-Cabello et al. 2009; van Hest and Tirrell 2001; Rabotyagova et al. 2011). Furthermore, with advances in recombinant DNA technology it is possible to design and produce tailored synthetic genes, allowing for the creation of multifunctional complex PBPs with total control over composition, molecular weight and structure.

Silk-elastin-like polymers (SELPs) are PBPs composed of multiple repeats of both silk- and elastin-like motifs that combine the outstanding mechanical and biological properties of both these proteins. Most commonly, they are based on repetitions of the silk fibroin amino acid motif GAGAGS and the mammalian elastin conserved motif VPGVG, with the proportions, number and sequence of these repeated motifs governing the properties of the polymer. The silk-like blocks spontaneously form hydrogen-bonded β-sheet crystals and impart thermal and chemical stability (Megeed et al. 2002) while the periodic inclusion of the elastomeric sequence reduces the overall crystallinity of the system and increases its flexibility and aqueous solubility (Cappello et al. 1990). These copolymers have already demonstrated their potential importance in several biomedical applications (Megeed et al. 2002; Gustafson and Ghandehari 2010) and more recently, in the fabrication of micro- and nano-structures (Nagarajan et al. 2007; Ner et al. 2009; Qiu et al. 2009). Nevertheless, to fully develop the potential of SELPs, new polymer designs with altered and improved properties are required, allowing for a better understanding of the design of these but also leading to an expanded range of novel polymers with potentially enhanced applicability.

In the present study we have designed and synthesised four novel SELPs in which the most commonly used elastin pentamer sequence VPGVG is altered to VPAVG. This simple substitution has been shown to significantly alter the mechanical properties of poly(VPGVG), changing its mechanical response from elastic to plastic deformation (Luan and Urry 1999; Nagapudi et al. 2005). Indeed, this material has unique properties among elastin-like polymers; its Young’s modulus is two orders of magnitude higher than that for poly(VPGVG) (Luan and Urry 1999) and while it does display a reversible inverse transition on heating (as do all other elastin-like polymers), this is found to be characterised by an acute hysteresis behaviour (Reguera et al. 2003; Machado et al. 2009). Hence, the preparation of SELPs based on combinations of this unique repetitive motif (VPAVG) with that of silk (GAGAGS) should give rise to a new set of polymers with expanded and potentially enhanced properties and applicability.

One of the principle limitations to the successful commercialisation of any recombinant protein or polymer is associated with the production and purification on a commercially viable level. SELPs are currently mainly produced in *Escherichia coli* using the *lacI* regulated T7 promoter-driven system by batch production in rich media, with volumetric productivities on the low miligram/L scale (i.e. approx. 20 mg/L) being reported (Nagarsekar et al. 2002; Haider et al. 2005; Dandu et al. 2009; Xia et al. 2011). Most commonly, the Sambrook protocol (Sambrook and Russell 2001) is used, with induction of protein production by the synthetic lactose analogue isopropyl-β-D-thiogalactopyranoside (IPTG) addition at the middle of the exponential growth phase. On the other hand, the use of auto-induction media whereby lactose added during the initial media preparation acts to automatically induce protein production (Studier 2005) and thereby circumvent the need for monitoring cell growth and addition of inducer, should allow for a more automated and efficient production method. Indeed, for high-throughput approaches this provides major advantages, avoiding intermediate steps during fermentation and minimising culture handling. In the present study, we examined the production of novel SELP copolymers with an auto-induction approach.

Purification of SELPs is most commonly carried out by immobilized metal affinity chromatography (IMAC) with affinity tags, in particular histidine-tags (6xHis) (Nagarsekar et al. 2002; Haider et al. 2005; Dandu et al. 2009; Xia et al. 2011). This approach is somewhat cumbersome however, requiring pretreatment steps such as cell disruption (e.g. by sonication) and separation of soluble cellular content (e.g. by centrifugation), and requiring the use of specialised and somewhat expensive matrices and equipment (Chow et al. 2008). In contrast, the use of non-chromatographic approaches can allow for a more economical, simplified and higher throughput purification process facilitating scale up to an industrial level (Chow et al. 2008). Indeed, the unique characteristics and known extreme chemical and thermal stability of fibrous proteins as well as of bio-engineered polymers based on these have been exploited in the development of simplified purification protocols for these. Elastin like polymers are frequently purified by temperature cycling, making use of their reversible inverse transition from soluble to insoluble form on heating above the inverse transition temperature (Meyer and Chilkoti 1999). High temperature treatment has been documented for the purification of recombinant spider silk protein (Scheller et al. 2001) and resilin-like polypeptides (Lyons et al. 2007). While these approaches are obviously advantageous as compared to affinity chromatography, they are nonetheless multistep processes requiring downstream processing and pretreatment. Moreover, they cannot be applied to our novel SELPs as temperature accelerates the irreversible gelation process (Haider et al. 2004). In contrast, the use of extremes of pH, and in particular acidic pHs at which fibrous proteins are known to be stable, may lead to an unfolding and precipitation of the host proteins and allow for both cell disruption and protein purification in one simple step. Here we examined the use of acidic pH combined with ammonium sulphate precipitation for a simplified downstream processing and purification protocol, thereby avoiding cumbersome cell disruption and chromatographic approaches and reducing process time and costs.

In this work, the genetic construction, development and optimisation of the autoinduction production protocol as well as a simplified non-chromatographic purification protocol for four novel SELPs (SELP-1020-A, SELP-520-A, SELP-59-A, SELP-109-A) is described and discussed.

## Materials and methods

### Materials

The cloning steps were performed in *Escherichia coli* strain XL1-Blue (Stratagene) and protein expression was achieved with *E*. *coli* strain BL21(DE3) (Novagen). Unless otherwise stated, chemicals were obtained from Panreac. Isopropyl β-D-1-thiogalactopyranoside (IPTG) was obtained from Formedium, tryptone from BD Biosciences and yeast extract from Cultimed, α-lactose was from BDH Chemicals and tris base from Fisher Scientific. Ethylenediamine tetraacetic acid disodium salt dihydrate (EDTA) and ampicillin were obtained from Sigma-Aldrich. Dodecyl sulphate sodium salt (SDS) and beta-mercapto-ethanol were obtained from Merck. HisTrap™ HP columns were from GE Healthcare. Restriction enzymes, T4 DNA Ligase, GeneRuler™ 1 kb DNA ladder and Taq Polymerase were obtained from Fermentas. Accuzyme Mix was obtained from Bioline and Antarctic phosphatase from NEB (New England Biolab). Plasmid extraction was performed with GenElute™ Plasmid Miniprep Kit (Sigma-Aldrich) and DNA purification from agarose gel by Nucleospin® Extract II (Macherey-Nagel). Cloning vector pDrive was obtained from Qiagen and modified as previously described (Rodriguez-Cabello et al. 2012). Expression vector pET-25b(+) was obtained from Novagen.

### Construction of SELP copolymers

**Figure 1:**
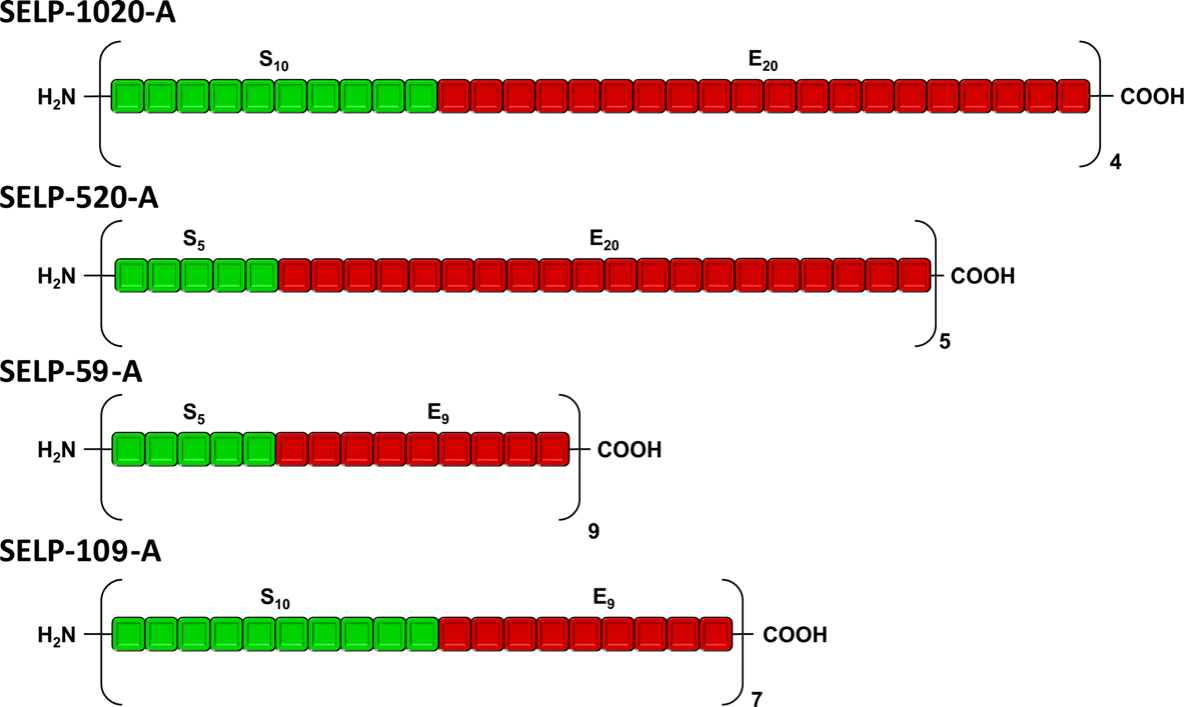
**Schematic representation of the block units for the four SELP copolymers, where Sx represents the number of silk-like blocks and Ey the number of elastin-like blocks.** Each hybrid block (SxEy) was self-ligated through a series of ligation reactions to achieve a number of tandem repeats corresponding to a molecular weight of approximately 55 kDa.

In this work we designed four different SELP copolymers with formulation (*SxEy*)*n*, where S represents the silk block with the sequence GAGAGS, *x*: the number of silk blocks; *E* represents the elastin-like block with the sequence VPAVG, *y*: the number of elastin-like blocks and *n* is the number of repetitions of the overall copolymer. All the hybrid copolymers were designed to have a similar molecular weight of 55 kDa but different silk to elastin block ratios or different number of silk blocks (Figure 1): SELP-1020-A with composition (S10E20)4; SELP-520-A with composition (S5E20)5; SELP-59-A with composition (S5E9)9 and SELP-109A with composition (S10E9)7. For nucleotide and amino acid sequences see [Additional file 1].

Genetic constructions were obtained by recursive directional ligation (Meyer and Chilkoti 2002; McDaniel et al. 2010) using previously described standard molecular biology procedures (Machado et al. 2009; Rodriguez-Cabello et al. 2012 codon usage while minimizing sequence repetition. All constructions and concatenations were performed in a modified pDrive cloning vector (Rodriguez-Cabello et al. 2012). The gene sequences were selected to emphasize *Escherichia coli*), transformed into *E*. *coli* strain XL1 Blue by the SEM method (Inoue et al. 1990) and confirmed by automated DNA sequencing. Vector dephosphorylation with antarctic phosphatase was performed prior to ligation with T4 DNA ligase. Synthetic DNA duplexes flanked by *Eam*1104I recognition sites and coding for 5 repetitions of the monomer GAGAGS (S5) were generated by polymerase chain reaction amplification with *Taq* polymerase. The elastin block was obtained by PCR amplification of two synthetic primers, VPAVG_FW_ with 173 nucleotides and VPAVG_REV_ with 171 nucleotides, flanked by *Eam*1104I recognition sites; this allowed obtaining the amplification of 9 and 11 repetitions of VPAVG (E9 and E11, respectively). Each amplicon was subsequently cloned into the modified pDrive cloning vector and selected by PCR colony screening. After cloning, each monomer was obtained by restriction digestion with *Eam*1104I and isolated and purified with a DNA gel extraction kit. To obtain the dimer S10, the monomer was subjected to self-ligation, while the block E20 was obtained by combination of E9 and E11. The hybrid polymers *SxEy* were constructed by ligation of the basic blocks and then subjected to concatenation ligation reactions to get the required *n* tandem repeats corresponding to the predicted molecular weight of about 55 kDa. The DNA sequence encoding each final SELP construct was digested with *Eam*1104I, blunt-ended and subcloned into pCM13 (a modified pET-25(+) expression vector). Blunt-ends were produced by filling the sticky-ends of DNA fragments with Accuzyme mix at 72°C for 30 minutes.

### Construction of expression vector pCM13

To allow for a correct insertion of all SELP encoding genes, the original pET-25b(+) expression vector was mutated by site-directed mutagenesis according to the method previously described by Ansaldi, M. *et al* (Ansaldi et al. 1996). The point mutations were introduced by PCR using the oligonucleotides described in Table 1.

**Table 1:** Primers pairs for introduction of selected mutations in pET-25b(+) expression vector

Pair #1	Reverse *Eco72* I	5^′^ GAGCAGCAGACCAGCAGCAGCGGTCGGCAGCA**C**GT**G**TT**C**CATATGTATATCTCCTT 3^′^
	Forward *Eco72* I	5^′^ CGCTGCTGCTGGTCTGCTGCTCCTCGCTGCCCAGCCGGC 3^′^
Pair #2	Reverse *Eco147* I	5^′^ GGGGTCTTCCGGGGCGAGTTCTGGCTGGCTAG**G**CC**T**TTTGATCTCGAGTGCGG 3^′^
	Forward *Eco147* I	5^′^ AGAACTCGCCCCGGAAGACCCCGAGGATGTCGAGCACCA 3^′^

Site-directed mutagenesis was performed by PCR with the Accuzyme Mix (high fidelity polymerase) (Queiros et al. 2001); initially by using pair #1 of primers and secondly by using pair #2 of primers (Table 1). Parental DNA was digested with *Dpn*I and PCR products were transformed to *E*. *coli* XL1 Blue. Point-mutations were confirmed by restriction digestion of plasmid DNA with *Eco*72 I for mutations introduced by pair #1, and *Eco*147 I for mutations introduced by pair #2, as well as by sequencing.

The aforementioned modified pET-25b(+), titled pCM13, was prepared for cloning by restriction digestion with the blunt-end producing enzymes, *Eco*72I and *Eco*147I, gel purified and dephosphorylated. This was then ligated with the end-filled SELP-1020-A, SELP-520-A, SELP-59-A and SELP-109-A DNA constructs. Insertion and correct orientation was evaluated by digestion with *Ppu*21I and automated DNA sequencing. The expression vectors pCM13(SELP-1020-A), pCM13(SELP-520-A), pCM13(SELP-59-A) and pCM13(SELP-109-A) were then transformed to *E*. *coli* BL21(DE3) for protein expression.

### Production in shake flasks

Overnight pre-cultures of 10 ml Lysogeny Broth (LB: 10 g tryptone, 5 g yeast extract, 5 g NaCl, per litre) were used to inoculate fresh cultures in shake flasks to an optical density of 0.01 (OD_600_) and allowed to grow for 24 hours. All the media used were supplemented with 150 μg/ml of ampicillin. Protein expression was analysed in LB with IPTG induction (0.5 and 1 mM); LB supplemented with 2 g/L of α-lactose (LB+Lac) to allow auto-induction; Terrific Broth (TB: 12 g tryptone, 24 g yeast extract, 0.17 M KH_2_PO_4_, 0.72 M K_2_HPO_4_, 5 g glycerol, per litre) with IPTG induction (0.5 and 1 mM) and TB supplemented with 2 g/L of α-lactose (TB+Lac) to allow auto-induction. Throughout this work, the term lactose will refer α-lactose. Other parameters considered include, temperature (30 and 37°C) and culture volume to flask volume ratio (1:2, 1:3, 1:4 and 1:5, corresponding to 500 ml, 333 ml, 250 ml and 200 ml of medium in 1 L Erlenmeyer flask). In cases where IPTG induction was used, cell cultures were allowed to grow to an OD_600_ of 1.0 and protein expression was induced by adding IPTG to a final concentration of 0.5 or 1 mM for 4 hours. In media supplemented with lactose, cells were allowed to grow for 24 hours. All the experiments were conducted in standard flat-bottomed glass Erlenmeyer 1 L flasks with cotton plugs and a standard 200 rpm agitation. Data represents mean values of at least 3 independent experiments.

### Analysis of protein production levels from cell crude extracts

For evaluation of protein production levels, samples of cell crude extracts were analysed by sodium dodecyl sulphate polyacrylamide gel electrophoresis (SDS-PAGE) and stained with copper chloride 0.3 M (Lee et al. 1987). Evaluation of protein expression levels in the different bacterial cell cultures was carried out by direct comparison between samples normalized for the same cell density. For normalisation, 1 ml of cell culture was collected, centrifuged and resuspended in 100 μl of TE buffer (50 mM Tris, 1 mM EDTA at pH 8.0) and supplemented with an additional 25 μl of sample loading buffer (10% w/v SDS, 10 mM beta-mercapto-ethanol, 20% v/v glycerol, 0.2 M Tris–HCl pH 6.8, 0.05% w/v bromophenol blue). Samples were kept at 4°C for 30 minutes and centrifuged at the same temperature for 20 minutes at 14,000×g. For electrophoresis, the volume of the total soluble cell crude extract (supernatant) to be applied in each lane was calculated according to the following formula: ODi/ODf = Vi/Vf, where ODi is the OD_600_ of the cell culture, ODf is 0.05 (this value was optimized for copper staining), Vi is 125 (100 μl of TE + 25 μl of sample loading buffer) and Vf is the volume of supernatant (μl) to apply. In the case of samples applied in the same gel, protein expression was visually estimated, supported by area calculation with the image processing software *ImageJ* (Rasband 1997–2011) and expressed as a relative percentage of the highest value. Whenever there was the need to compare expression levels in different gels, analysis was carried out by visually comparing band intensity/thickness. The same volume of 3 μl of molecular weight marker was used in every gel. No modifications were made to the images other than cutting, pasting and resizing.

### Protein production and purification

For all SELP copolymers, bacterial cell cultures were grown in TB+lac containing ampicillin, with a liquid to flask volume ratio of 1:5 (200 ml of medium in 1 L flask) at 37°C. After fermentation time, cells were harvested by centrifugation and frozen until use. For cell lysis, cells were thawed, resuspended in the adequate buffer and lysed by ultrasonic disruption (sonication) using a Vibra cell™ 75043 (Bioblock Scientific, 750 W max power) with a solid probe of 25 mm diameter. Ultrasonic treatment was applied with an amplitude of 65% for 3 seconds, followed by a 9 second pulse-off delay with a total sonication time of 30 minutes. Samples were kept on ice throughout the process. Following disruption, cellular debris was removed by centrifugation at 11,500×g for 20 minutes at 4°C.

Recombinant SELP-1020-A was purified by immobilized metal affinity chromatography (IMAC) and compared with the combined acid pH treatment and ammonium sulphate precipitation. For all the remaining copolymers, purification was obtained by the combined purification method. Protein purity, molecular weight and recovery rates were assessed by SDS-PAGE with copper chloride (0.3 M) staining at each protein purification stage.

#### Purification by immobilized metal affinity chromatography (IMAC)

Recombinant protein from SELP-1020-A culture was purified by IMAC with a HisTrap HP 5-ml column already prepacked with a Ni Sepharose™ High Performance matrix. The cells pellet was thawed, resuspended in binding buffer (20 mM sodium phosphate, 0.5 M NaCl, 20 mM imidazole, pH 7.4) and lysed by ultrasonic disruption (sonication) as described above. The insoluble debris was removed by centrifugation and the clear supernatant filtered through a 0.45 μm filter to avoid column clogging. Elution was performed with buffers containing 0.5 M NaCl, 20 mM phosphate buffer, pH 7.4 and increasing concentrations of imidazole (20 mM, 40 mM, 80 mM, 150 mM, 200 mM, 250 mM and 500 mM), by following the manufacturer’s instructions. The fractions were analysed for SELP content by SDS-PAGE with copper chloride staining. Final polymer purification from the soluble fraction was performed with buffer containing 80 mM imidazole, 0.5 M NaCl and 20 mM phosphate buffer, pH 7.4.

#### Acid pH treatment

Cultures with pCM13(SELP-1020-A)-BL21(DE3) were grown in the previously optimised conditions for 22 hours. Cells were collected, resuspended in TE buffer (50 mM Tris, 1 mM EDTA, pH 8) and lysed by sonication as described above. The insoluble debris was removed by centrifugation and the supernatant adjusted to pH 4, 3.5 or 3 with 1 M hydrochloric acid (HCl), while maintaining on ice with constant agitation. After an incubation period of 30 minutes at the corresponding pH, the precipitated supernatant was centrifuged at 11,500×g for 20 minutes at 4°C. The insoluble debris was removed and the clear supernatant analysed by SDS-PAGE.

#### Ammonium sulphate precipitation

Cell pellets were thawed and resuspended in TE buffer and lysed by sonication as described above. The insoluble debris was removed by centrifugation and the clear supernatant was adjusted to pH 3.5 with HCl 1 M, leading to precipitation of most of the *E*. *coli* proteins. The precipitated proteins were removed by centrifugation at 11,500×g for 20 minutes at 4°C. To assess the optimal concentration of ammonium sulphate for purification of recombinant SELP copolymers, the supernatant of each recombinant protein (SELP-1020-A, SELP-520-A, SELP-59-A and SELP-109-A) was precipitated with ammonium sulphate at final concentrations of 10, 15, 20, 25, 30, 35 and 40% saturation. Ammonium sulphate was added slowly to the supernatant while maintaining on ice with agitation and the resulting mixture was kept on ice for 30 minutes prior to centrifugation at 11,500×g for 20 minutes at 4°C. After centrifugation, the precipitated polymer fraction was resolubilized in deionized water for 2–3 hours at 4°C and centrifuged or filtered to remove the remaining insoluble contents. The clear polymer solution was then dialysed against water at 4°C, filtered with a 0.45 μm filter and lyophilized.

#### Evaluation of cell disruption by acidic pH treatment

For evaluation of the efficiency of the acidic pH treatment for cell disruption, SELP-59-A producing cultures were used. Cells were collected, resuspended in TE buffer at final wet cell weights of 0.4 g/ml and 0.8 g/ml and subjected to acid treatment or sonication as described above. For the acid treatment, suspensions were adjusted to pH 3.5 with 1 M HCl and incubated overnight at 4°C with mild agitation (acid-based cell lysis). Percentage of cell survival was compared in cells submitted to 1) acid-based cell lysis, 2) sonication, 3) acid-based cell lysis followed by sonication and 4) untreated cell crude extract. After each treatment, samples were taken, plated on LB agar without antibiotic at appropriate dilutions (10^-7^ for the untreated sample and 10^-6^ for the remaining) and incubated overnight at 37°C. Cells were counted and the results expressed as percentage of cell survival compared to the untreated sample. All experiments were done in triplicate. Evaluation of protein release was conducted in cell extracts submitted to acid-based cell lysis and acid-based cell lysis followed by sonication. After each treatment the cell extract was centrifuged for 20 minutes at 11,500×g and the supernatant was collected for further analysis by SDS-PAGE. The untreated cell extract was prepared according to the method previously described in Protein Analysis in Material and Methods. The same volume of cell extract/sample was used for all experiments as well as for SDS-PAGE.

## Results

### Optimization of culture conditions

**Figure 2:**
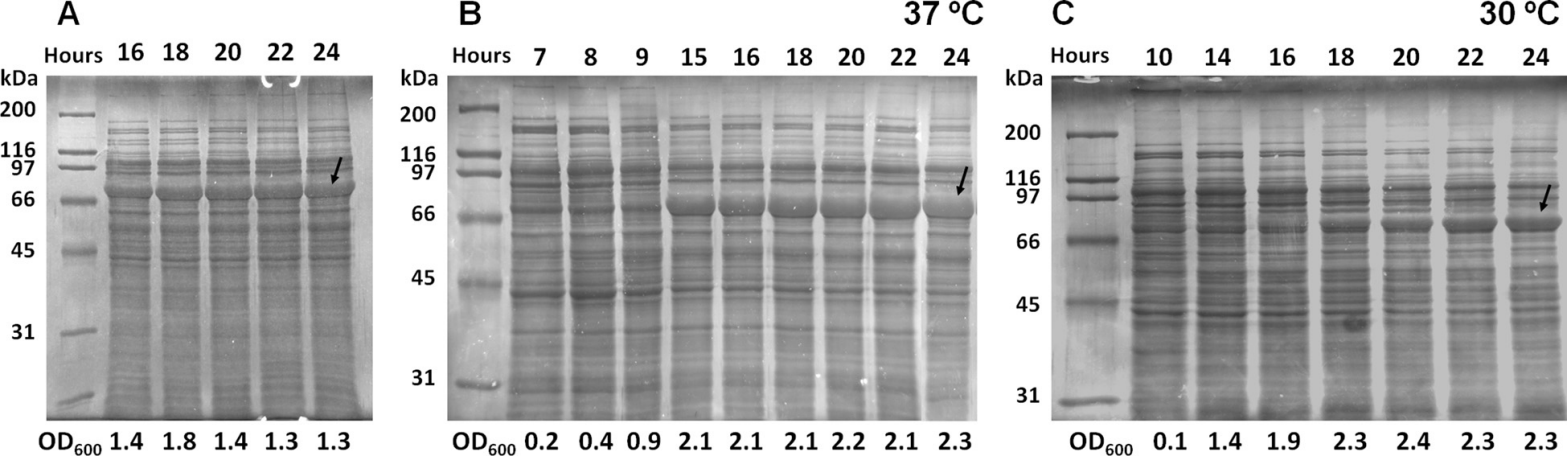
**Electrophoretic patterns of cell crude extracts during growth of auto-induced pCM13(SELP-1020-A)-BL21(DE3) cultures in A) LB+lac at 37°C, B) TB+lac at 37°C and C) TB+lac at 30°C.** To facilitate direct comparison of protein expression, all samples were normalised for the same cell density before loading on gel. The sampling time is given above each lane whereas the OD600 at that time point is indicated below. Target recombinant protein is indicated by arrows and the molecular weight marker is in the left of each gel. The abnormal gel mobility of the recombinant protein was previously observed by other authors (Teng et al. 2009; Lyons et al. 2007; McPherson et al. 1992).

*E*. *coli* BL21 (DE3) transformed with pCM13(SELP-1020-A) was chosen as the representative sample for production optimization studies. Initial studies aiming at protein production were performed in LB and TB media supplemented with lactose (LB+lac and TB+lac), at 37°C with a liquid to flask volume ratio of 1:5 and analysed through a 24 hour fermentation period. Figure 2 represents the cell crude extract of protein samples taken at different time periods during the 24 hour shake flask fermentation, showing that higher ODs (1.3 for LB+lac in Figure 2A and 2.3 for TB+lac in Figure 2B) as well as higher production levels could be obtained with TB supplemented with lactose at each time period.

Visually, SELP-1020-A expression levels seem to reach a steady state after 18 hours of fermentation in LB+lac (Figure 2A) which was further corroborated by calculating the area of the protein bands (Table 2). Regarding SELP-1020-A production at 37°C in TB+lac, protein expression starts in the period between 9 and 15 hours of fermentation and reaches a steady state after 18 hours (Table 2) with little variation in protein band area or cell density. When the temperature of the bacterial cell culture was reduced to 30°C, while maintaining all the other conditions (TB+lac, agitation and flask volume ratio), protein expression was delayed, starting only after 16 hours of fermentation and showing lower protein expression (Figure 2C). In this case, protein expression increased gradually over time as determined by calculating the area of the expressed SELP-1020-A band (Table 2. For SDS-PAGE analysis, samples were reverse stained with copper chloride according to the method previously described by Lee *et al.* (Lee et al. 1987). This staining method was preferred over conventional Coomassie blue staining not only due to the greater sensitivity (≈10 ng protein/lane) but also due to the poor Coomassie staining of SELP-1020-A bands that appear extremely diffuse and make band analysis difficult. Although copper staining can be a coarse method for comparing samples in different gels, by applying normalised samples for the same cell density, evaluation of protein expression levels can be directly compared on the basis of the protein band thickness.

**Table 2:** Relative productivities of SELP-1020-A as determined by area calculation of protein bands

	**Relative percentage (%)**						
	**LB+lac 37°C**	**TB+lac 37°C**	**TB+lac 30°C**	**Flask volume ratios (TB+lac, 37°C)**			
				**1:2**	**1:3**	**1:4**	**1:5**
15 hours	n.d.	89	n.d.	n.d.	n.d.	n.d.	n.d.
16 hours	86	87	37	n.d.	n.d.	n.d.	n.d.
18 hours	100	100	50	n.d.	n.d.	n.d.	n.d.
20 hours	99	96	67	n.d.	n.d.	n.d.	n.d.
22 hours	93	99	91	100	99	100	98
24 hours	95	97	100	n.d.	n.d.	n.d.	n.d.

Bacterial cells were cultured in LB+lac at 37°C, TB+lac at 37°C, TB+lac at 30°C and TB+lac at 37°C with different liquid to flask volume ratios (1:2, 1:3, 1:4 and 1:5). Band areas were calculated with ImageJ and expressed as relative percentage. n.d. – not determined.

**Figure 3:**
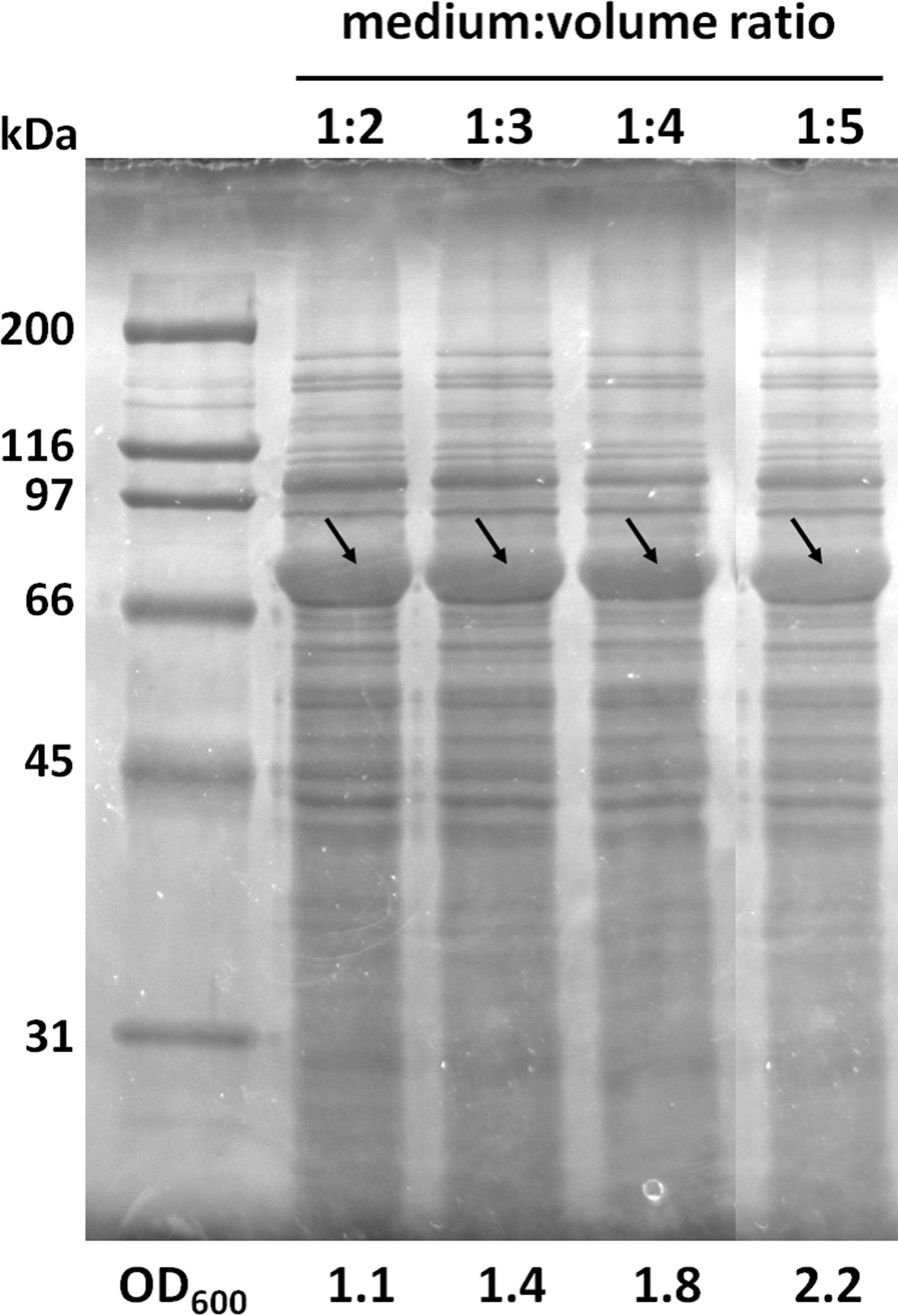
**Cell growth and SELP-1020-A expression using auto-induction medium with different flask volume ratios after 22 hours of fermentation.** All samples were normalised for the same cell density prior to loading on gel. This figure was assembled from pictures of different gels. No modifications were made to the images other than cutting, pasting and resizing. Cell density is indicated below each gel with target recombinant protein indicated by arrows. Molecular weight marker is represented on the right and left lanes.

In shake flasks, the oxygen transfer rate (OTR) is reversely proportional to the volume of culture (Maier and Büchs 2001). Higher OTR is obtained in flasks with less culture volume or by increasing the agitation rate, however one should take in account that very low amounts can only be used for short-term fermentations otherwise the medium will evaporate. To study the effect of OTR in SELP-1020-A production, a preliminary study of protein expression with different liquid to flask volume ratios was carried out with the previously optimised conditions (TB+lac, 22 hours fermentation at 37°C). Considering SELP-1020-A, high protein expression levels with comparable band intensities were obtained for all the ratios investigated (Figure 3). The relative percentage of protein production is summarized in Table 2 demonstrating that for all the volume ratios tested there was little or no variation.

Regarding bacterial cell growth, lower volume of medium in the flasks resulted in higher cell density as determined by the OD_600_. For all the cases, the OD increased with increasing volume ratios with values of 1.1, 1.4, 1.8 and 2.2 for volume ratios of 1:2, 1:3, 1:4 and 1:5, respectively. For the subsequent production of all SELP copolymers the standard parameters were set as a 22 hour fermentation period in TB+lac, with a flask volume ratio of 1:5 (200 ml of medium in 1 L flask) at 37°C.

### SELP-1020-A production with IPTG induction

**Figure 4:**
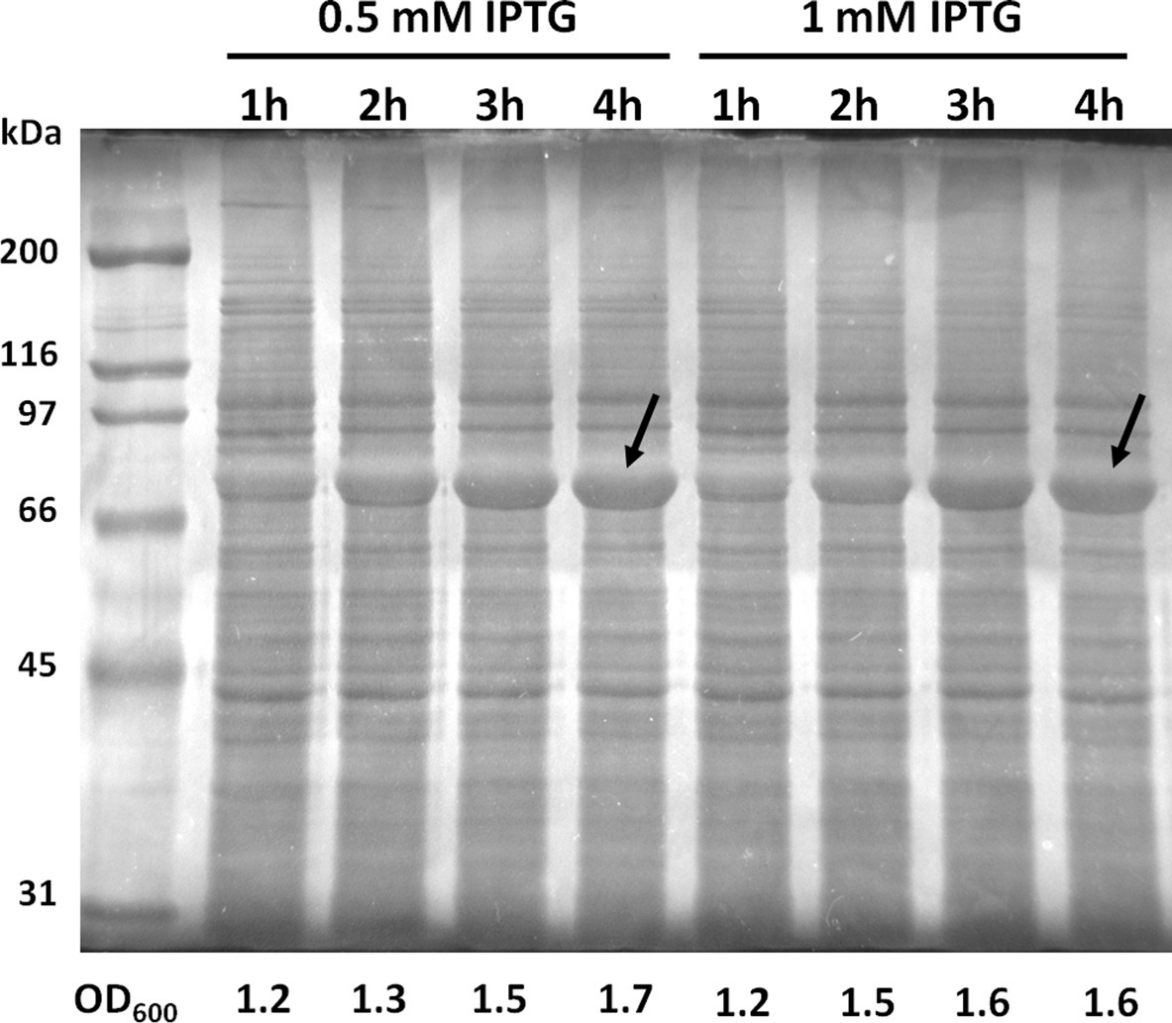
**Expression levels with respect to the concentration of inducer and time course of induction.** Cultures of *E*. *coli* harboring pCM13(SELP-1020-A) in LB were induced with 0.5 mM or 1 mM of IPTG when the OD_600_ reached 1. Samplings were taken at the indicated intervals. Cell density is indicated below each lane with target recombinant protein indicated by arrows.

As the reference model of induction, we have chosen the widely used protocol for recombinant protein expression described in both, the Sambrook laboratory (Sambrook and Russell 2001) and the pET system (Novagen 2006) manuals. In cell cultures in LB medium, for the two concentrations of IPTG tested (0.5 mM and 1 mM), the best expression levels were obtained after 4 hours of induction, with similar band intensities for both concentrations (Figure 4). Comparable results with similar expression levels were obtained with TB (data not shown) however, and as expected, the final optical density reached higher values (approximately 2.4 for TB and 1.6 for LB for both concentrations of IPTG).

### Copolymers purification

**Figure 5:**
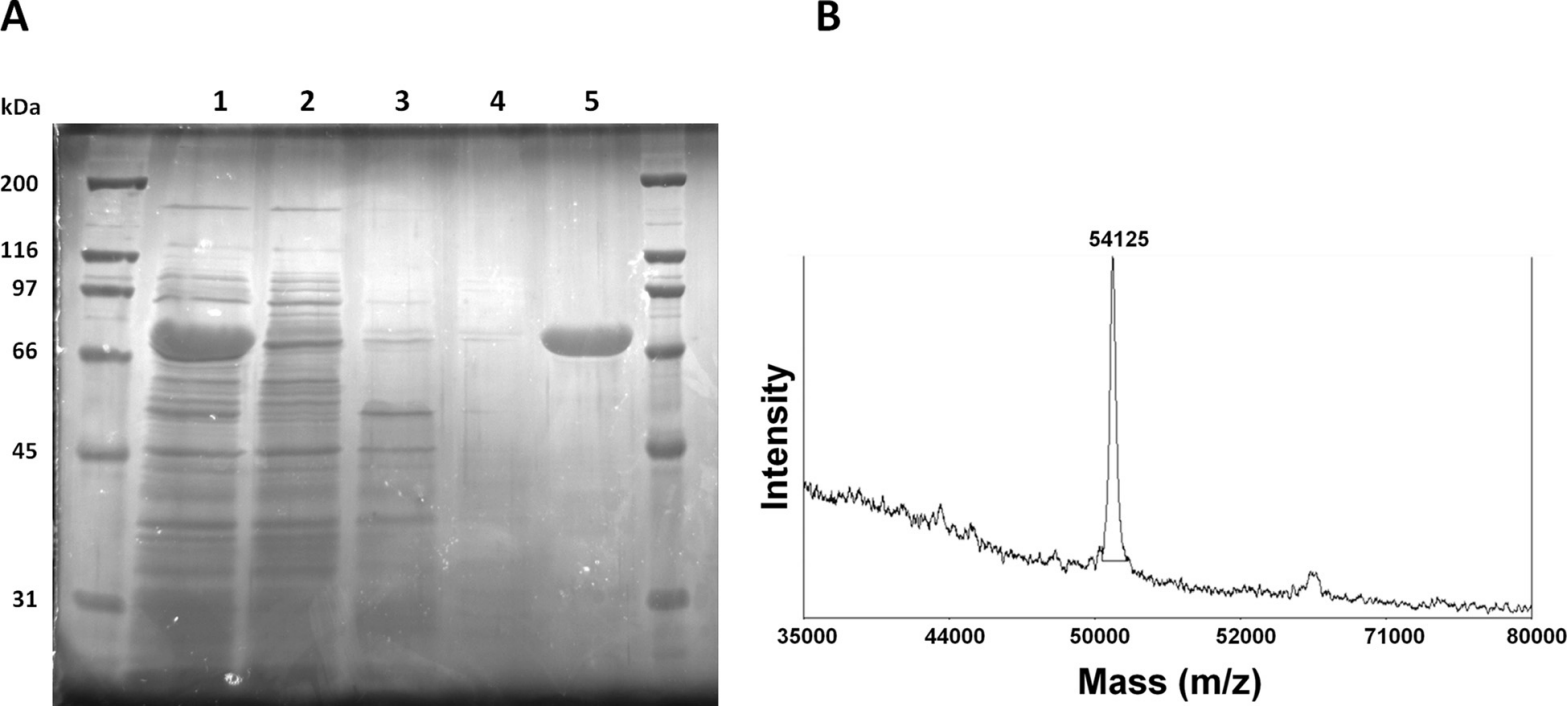
**Purification of SELP-1020-A by A) affinity chromatography and B) molecular weight determination by mass spectrometry. A)** SDS-PAGE of SELP-1020-A: lane 1 – soluble lysate before column loading; lane 2 – flow through; lane 3 – fraction eluted with 20 mM imidazole; lane 4 – fraction eluted with 40 mM imidazole; lane 5 – fraction eluted with 80 mM imidazole. Fully purified polymer fraction was obtained by elution with buffer containing 80 mM of imidazole with high recovery rate. **B)** Image represents the MALDI-TOF mass spectrum of SELP-1020-A. The molecular ion peak at 54125 confirms the theoretical calculated value (54117 Da).

During the optimisation of culture conditions for protein production, we realized that SELP-1020-A was expressed in the soluble form and allowing for purification by IMAC. SELP-1020-A was expressed in TB+lac, in the conditions here reported and cells were treated as described in the Materials and Methods section. The recombinant polymer was purified from the clear supernatant with the HisTrap HP nickel column using sequential elutions with buffers containing imidazole. A highly purified, as indicated by SDS-PAGE analysis, SELP-1020-A polymer fraction was obtained by eluting with buffer containing 80 mM of imidazole (Figure 5). Little or no SELP-1020-A losses were observed during the process and no recombinant protein was present in further elutions with higher concentrations of imidazole (150 mM, 200 mM, 250 mM and 500 mM), showing that SELP-1020-A was purified with a high recovery rate. However, an apparent molecular weight of approximately 66 kDa, determined from the SDS-PAGE analysis, was much higher than the theoretical calculation of 54117 Da for the same protein. The unexpected higher apparent molecular weight, as determined by SDS-PAGE, was previously observed by other authors (Teng et al. 2009; Lyons et al. 2007; McPherson et al. 1992) and attributed to the hydrophobic nature of these protein polymers. MALDI-TOF MS was therefore used to compare the molecular weight of the chromatographically purified copolymer and showed a molecular ion peak at 54125 Da, which is in agreement within experimental error, with the theoretically calculated value of 54117 Da (Figure 5B).

Although being a popular and generally used method for purification of recombinant proteins, immobilised metal-affinity chromatography has serious limitations, namely the difficulties associated with scale-up, high cost and column fouling. The use of non-chromatographic methods would overcome these drawbacks and hence we investigated acid pH treatment and ammonium sulphate precipitation as methods for SELP purification. Indeed the latter approach has already been applied successfully as part of a purification protocol for recombinant spider silk protein (Scheller et al. 2001) and resilin-like proteins (Lyons et al. 2007; Kim et al. 2007). These alternative purification methods are less labour intensive and less expensive and should allow for high recovery rates of recombinant proteins known to be stable under the conditions used. The low pH would be expected to precipitate the endogenous *E*. *coli* proteins while the more stable SELPs would stay in solution.

**Figure 6:**
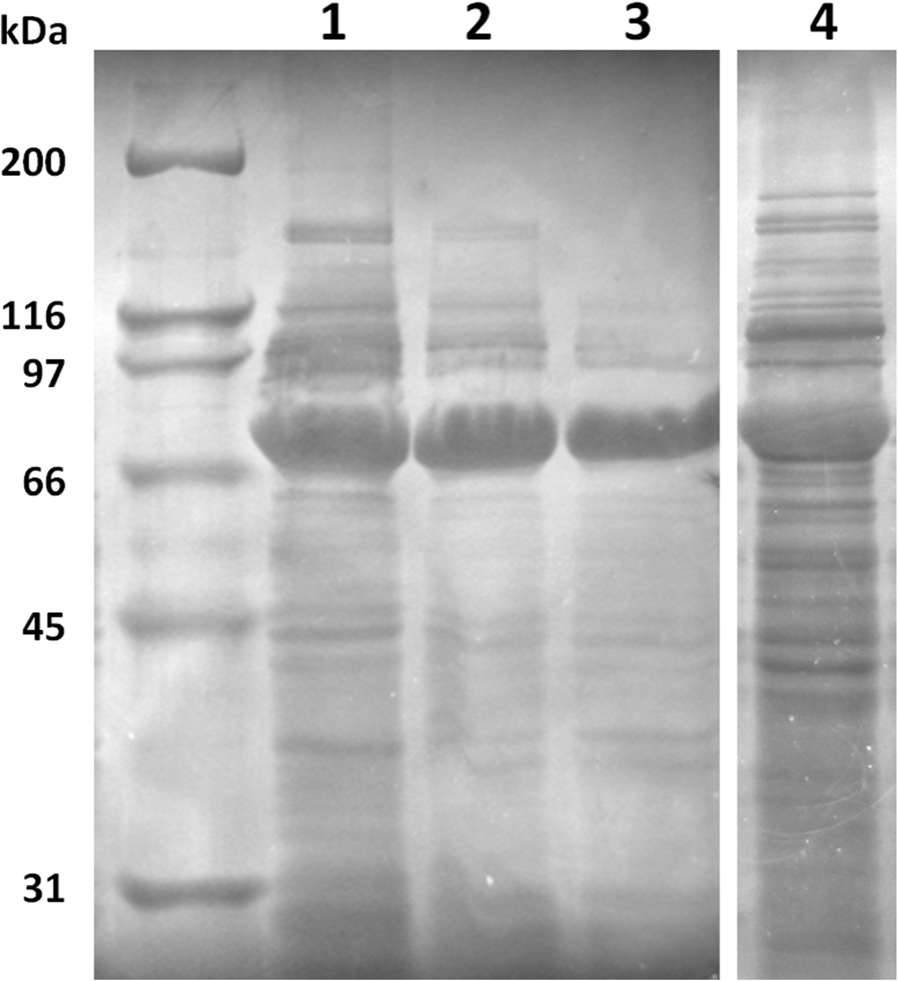
**Effect of the acidic treatment of the soluble lysate.** The clear supernatant of the lysate from SELP-1020-A (lane 4) was adjusted to pH 4 (lane 1), 3.5 (lane 2) and 3 (lane 3). After pH adjustment, the precipitated pellet was removed and the clear supernatant was analysed by SDS-PAGE. Nearly pure SELP-1020-A was obtained after the acidic treatment at pH 3.

Bacterial cells harbouring pCM13(SELP-1020-A) were allowed to grow in the previously optimised conditions, collected and lysed. After cell disruption, the insoluble debris was removed by centrifugation and the supernatant was adjusted to pH 4, 3.5 or 3. While adjusting the pH, the precipitation of proteins was clearly visible with the solution turning opaque with a whitish colour. After removal of precipitated proteins, the clear supernatant was analysed by SDS-PAGE, showing that adjusting the pH of the crude lysate to 3.0 or 3.5 was very effective in the removal of *E*. *coli* proteins (Figure 6). Treatment at pH 3.0 demonstrated to be more effective in the removal of contaminant proteins. However, the lower intensity of the band suggests that there is a loss of the recombinant copolymer during the process, which is probably due to a partial precipitation. Therefore, treatment at pH 3.5 was apparently more efficient for purification, without detectable SELP losses being observed during the process. In the case of SELP-1020-A, adjusting the pH of the crude lysate to 3.0 was almost enough for purification of the copolymer. However, purification with only the acidic treatment was not very reliable as in some random rare occasions the supernatant presented some contaminant proteins, although in very low amounts. In the case of SELP-59-A and SELP-520-A some contaminants do remain after acidic pH treatment and therefore ammonium sulphate precipitation was examined as a further non-chromatographic step for completion of purification.

**Figure 7:**
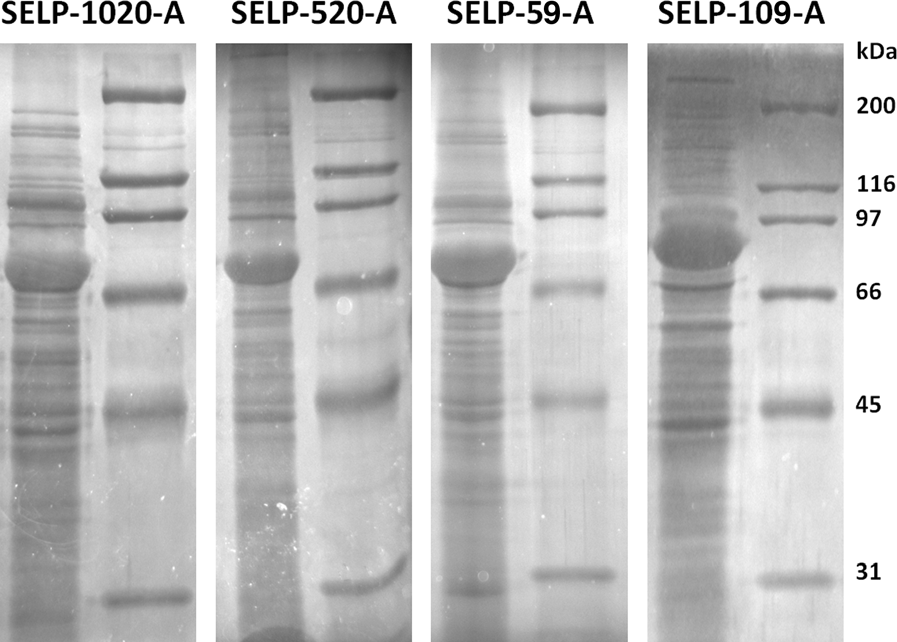
**Cell crude extract obtained from bacterial cell cultures producing the different copolymers.** This figure was assembled from pictures of different gels. No modifications were made to the images other than cutting, pasting and resizing.

All the recombinant SELPs were produced in the optimised conditions. SDS-PAGE analysis of the cell crude extracts revealed that high levels of protein expression were obtained for all the recombinant copolymers (Figure 7). Although showing a good protein expression as mentioned above, no further attempts were made for the purification of SELP-109-A as its precipitation occurred even during cell lysis (data not shown).

**Figure 8:**
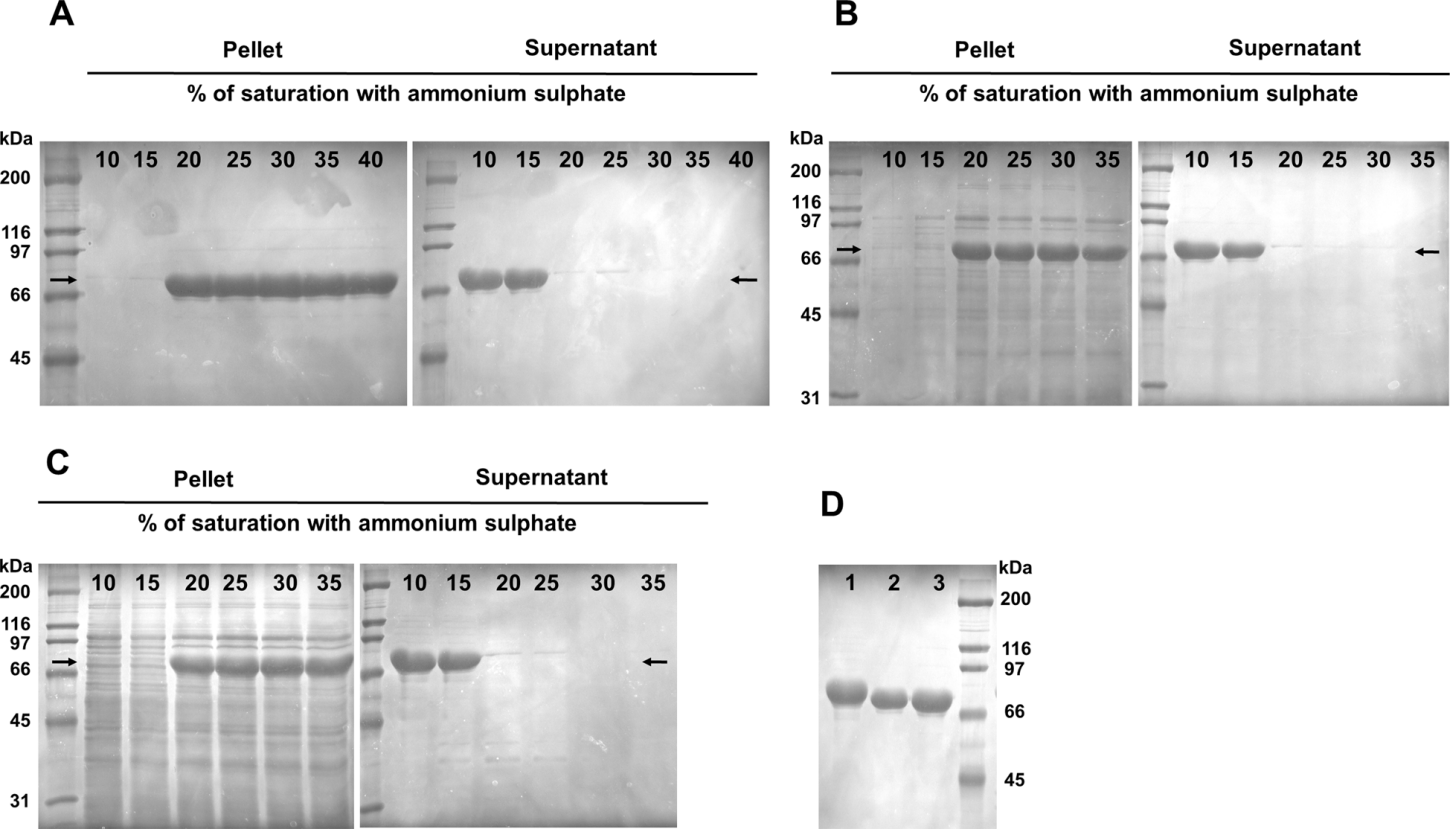
**Purification of the recombinant copolymers by ammonium sulphate precipitation.** The soluble acid-treated lysates of SELP-1020-A **(A)**, SELP-520-A **(B)** and SELP-59-A **(C)** were saturated with increasing concentrations of ammonium sulphate (indicated above each gel). Depending on the concentration used, the copolymers either precipitated or stayed in the supernatant. **(D)** ( Increased purity was obtained by resuspending the precipitated copolymer (with 20% ammonium sulphate) in water and letting at 4°C with agitation followed by centrifugation or filtration (lane 1 – SELP-1020-A, lane 2 – SELP-520-A, lane 3 – SELP-59-A). The abnormal gel mobility of the recombinant protein was previously observed by other authors (Teng et al. 2009; Lyons et al. 2007; McPherson et al. 1992) and attributed to the hydrophobic nature of the proteins.

Due to the hydrophobic nature of SELPs, all the remaining copolymers (SELP-1020-A, SELP-520-A and SELP-59-A) precipitated from the acid-treated lysate at relatively low concentrations of ammonium sulphate. For all the copolymers, 20% saturation was optimal for precipitation from the soluble lysate with most of the *E*. *coli* endogenous proteins being retained in the supernatant (Figure 8).

Figure 8A shows the fractions (pellets and supernatants) resulting from the precipitation of SELP-1020-A acid-treated extracts with increasing concentrations of ammonium sulphate. Due to the treatment with pH, the polymers, and in particular SELP-1020-A, were already highly pure. Precipitation with 20% ammonium sulphate allowed for both purification and concentration of the samples and in this way avoided high volumes for dialysis and subsequent lyophilisation. Furthermore, no protein losses were detected by SDS-PAGE, indicating high recovery rates of recombinant protein.

Finally, increased purity of the three copolymers was obtained by resuspending the precipitates in deionized water for 2–3 hours at 4°C with agitation. The recombinant copolymers were readily dissolved in the aqueous solution while the *E*. *coli* contaminants remained in the insoluble state. Removal of the insoluble contaminant debris was achieved by centrifugation and further filtration allowing for obtaining of highly pure polymer fraction (Figure 8D).

After lyophilisation, the volumetric productivity (milligrams of recombinant protein per litre of culture) of the recombinant copolymers produced in the optimised conditions and purified by ammonium sulphate precipitation were 185, 151 and 198 mg/L for SELP-1020-A, SELP-520-A and SELP-59-A, respectively.

#### Acid-based cell lysis and protein release

To further simplify the purification protocol, we have investigated if an acid-based cell lysis could be employed as a single step method for both cell disruption and removal of contaminant *E*. *coli* proteins. To assess if bacterial cells were disrupted by the acidic treatment, therefore releasing the SELP copolymer, we measured the content of protein release by SDS-PAGE. Cell death was analysed by counting the number of colony-forming units (CFUs), and compared with the untreated cell crude extract.

**Figure 9:**
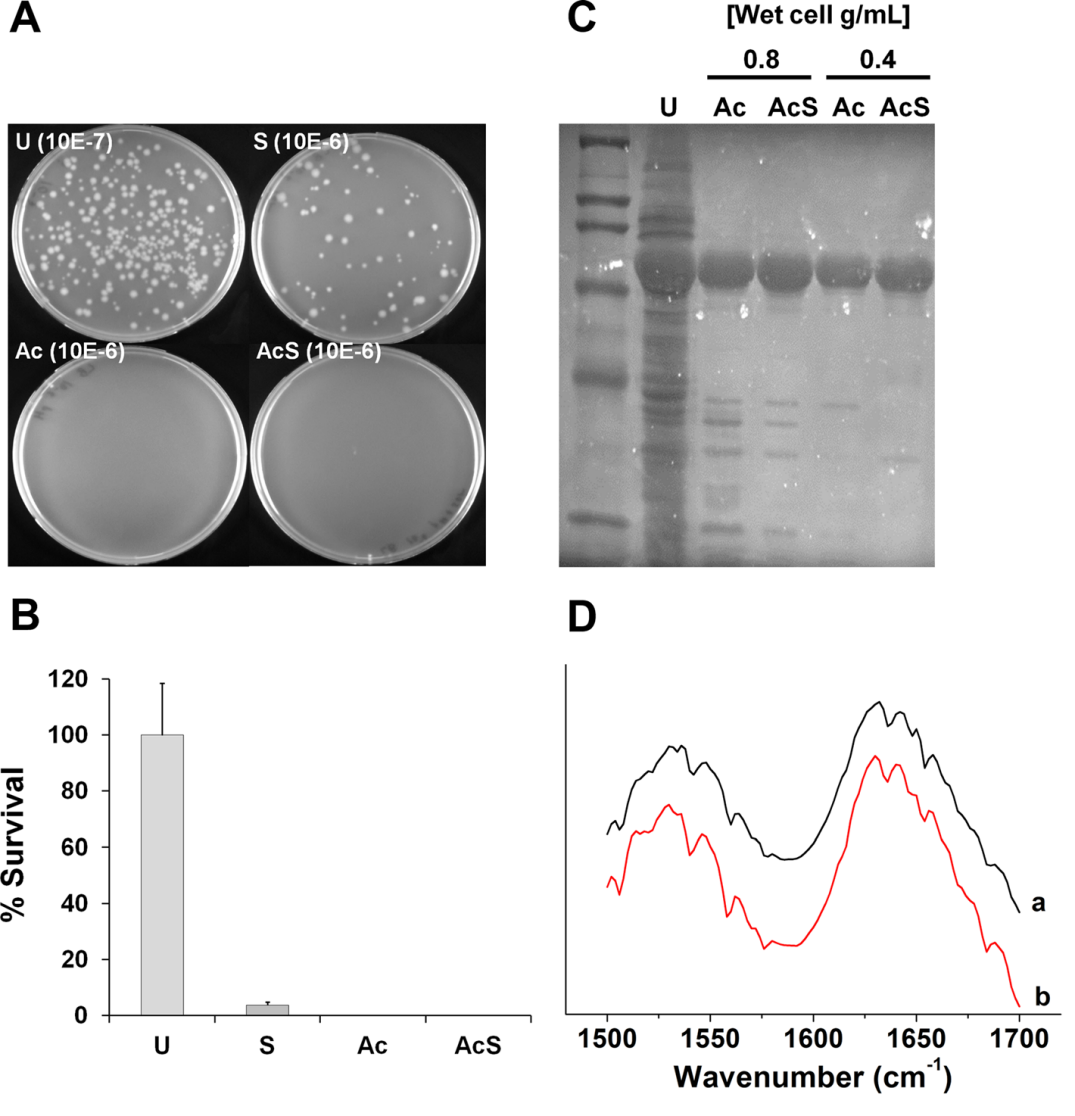
**Evaluation of cell death and protein release in bacterial cultures of SELP-59-A. (A)** – number of CFUs in untreated cell crude extracts (U) and in cell extracts submitted to sonication (S), acid-based cell lysis (Ac) and acid-based cell lysis followed by sonication (AcS). Cell dilutions are represented in parenthesis. **(B)** – Graphic representation of the percentage of cell survival for the different treatments with results expressed as percentage of survival as compared to the untreated sample. **(C)** – Evaluation of protein release in samples at different concentrations of wet cell weight after acid-based cell lysis (Ac) and acid-based cell lysis followed by sonication (AcS). The same volume was applied in each lane. Samples legend: U – untreated cell crude extract; Ac – acid-based cell lysis; AcS – acid-based cell lysis followed by sonication. **(D)** – FTIR spectra displaying the amide I (1600 – 1700 cm-1) and amide II (1500 – 1600 cm-1) band regions of pure lyophilized SELP-59-A samples without **(a)** and with **(b)** the acidic treatment.

As can be seen in Figures 9A and 9B, only about 4% cell survival was observed after sonication, represented by a low number of colony-forming units (CFUs). However, the acid-based cell lysis proved to be even more efficient as no CFUs were detected in solid medium, thus representing 0% cell survival. As expected, no CFUs were formed in the sample submitted to both acidification and sonication. In terms of protein release, the acid-based cell lysis proved to be very efficient at the two concentrations tested, showing that up to 0.8 g/ml of wet cell weight can be successfully disrupted by this method (Figure 9C). In fact, the supernatants of the treated samples exhibit similar levels of recombinant copolymer as that present in the cell crude extract while also allowing for the removal of most of the *E*. *coli* endogenous proteins. The additional sonication treatment did not influence the amount of protein in the supernatant of the acid-lysate, thus suggesting that a sole acidification step is enough to promote cell lysis and partial protein purification. Increased purity can then be achieved by further purification with ammonium sulphate as described above.

In order to evaluate if the acidic treatment would induce any conformational changes in the molecular structure of the copolymers, Fourier transform infrared spectroscopy (FTIR) was employed to assess possible structural changes between samples purified with and without the acidic treatment. Figure 9D shows the FTIR spectra in the amide I (1600 – 1700 cm^-1^) and amide II (1500 – 1600 cm^-1^) regions of pure lyophilized SELP-59-A samples, demonstrating that the acidic treatment did not induce significant structural modifications.

## Discussion

In this study we successfully synthesised and overexpressed four novel SELPs of similar size (55 kDa) but with variable content and ratios of silk to elastin i.e. 1:1 (SELP-109-A), 1:2 (SELP-1020-A, SELP-59-A) and 1:4 (SELP-520-A). Due to the novel elastin block (VPAVG) and the various silk to elastin ratios used processing of these polymers should give rise to a novel family of SELPs with unique biological and mechanical properties and thereby potentially extend the functionality of SELPs in general. Indeed, those polymers with a higher proportion of the elastin-like motif may be expected to be characterised by a reduced tensile strength, higher resilience, lower propensity for hydrogel formation, increased solubility and reduced crystallinity as compared to those with a higher proportion of silk (Dandu et al. 2009; Gustafson and Ghandehari 2010; Haider et al. 2005). The four polymers prepared in this study have been tailored to cover a large range of many of these properties and studies are currently underway to characterise these.

We examined the expression of these polymers with the currently most used method (Sambrook and Russell 2001) and found all proteins to be expressed intracellularly in a soluble form. In an attempt to simplify the production protocol we investigated the use of an auto-induction approach in which the inducing agent (in this case lactose) was added directly during media formulation. The best expression levels were obtained with TB+lac at 37°C when compared with LB+lac at the same temperature. However, despite the high expression levels, low cell densities (OD_600_ ≈ 2) were reached. This can be further optimised by adding low concentrations of glucose as previously demonstrated by Studier (Studier 2005), where the addition of glucose at low amounts (0.05% w/v) allowed cells to have an initial burst in growth. However, it should be noted that addition of glucose causes saturated cultures to become very acidic, limiting saturation density (Studier 2005). In this study we have chosen not to add glucose to medium composition, as our intention was to perform a preliminary study in the evaluation of protein expression levels where expression is induced in the early stages of growth. It is well known that the presence of glucose exerts catabolite repression, preventing the uptake and utilization of lactose, whilst glycerol does not interfere with lactose induction or the ability to metabolize this sugar for energy. In fact, addition of glycerol in auto-induction media was demonstrated to double the yield of target recombinant protein when compared to medium relying on lactose as the primary energy source. We have found that expression of the recombinant copolymers only occurs in the period between 9 to 15 hours of growth with no recombinant protein being produced earlier. This delay in the induction of protein expression is most likely attributed to the yeast extract used in the complex media formulation. The yeast extract supplies a variety of metabolites including complex carbohydrates that allow for fast initial growth rates. However, the yeast extract is also rich in amino acids that may prevent induction of target proteins by lactose in the early stages of growth. Indeed, in the absence of glucose, the presence of amino acids appears to inhibit induction until growth slows upon approach to saturation (Studier 2005).

One of the major factors affecting both bacterial cell growth and recombinant protein expression is the concentration of dissolved oxygen in the medium. It is commonly accepted, that high oxygen transfer rates lead to higher cell densities in bacterial cell cultures (Losen et al. 2004, Collins et al. *unpublished*). In shake flask fermentations, the OTR is influenced by several factors namely shaking frequency and shaking diameter, filling volume, flask shape and size, surface properties of the flask material and the physical-chemical properties of the liquid (Maier and Büchs 2001). Increasing the shaking frequency or lowering the filling volume leads to higher OTR due to liquid mixing and a high area of contact that allows efficient oxygen diffusion (Maier and Büchs 2001; McDaniel et al. 1965; McDaniel and Bailey 1969). In this work, we have performed a preliminary study on the effect of oxygen on the recombinant protein expression by evaluating the expression levels in shake flask cultures with different liquid to flask volume ratios. Cell density was shown to be inversely proportional to the filling volume without any deficiencies in protein expression (Figure 3) and thus suggesting that an optimization of oxygen levels could play a major factor in improving cell densities.

Auto-induction is more convenient than IPTG induction as it does not require any intermediate steps. The expression host is simply inoculated in the medium and let to grow without the need to monitor cell culture and add inducer at the appropriate time. In our study, induction of protein expression by lactose showed similar or even higher expression levels when compared with the conventional IPTG induction method. This work provides an insight into small/laboratory scale fermentations where auto-induction can provide major advantages as no extra culture handling is required. All copolymers were found to be successfully overexpressed in this auto-induction media, demonstrating its high potential as a facile method for production of recombinant SELPs in small/laboratory scale fermentations, but also for the production of several other proteins. In fact, in our laboratory, several other recombinant proteins were produced by this auto-induction method and purified for biotechnological purposes, namely, structural protein-based polymers, human growth factors, serine proteases, hydrolases, esterases, lipocalines, antimicrobial peptides and plant transcription factors (*personal communication*, *Casal, M*.). In all the studies performed above we used purified lactose but other sources of lactose can be used. Waste pollutants derived from the dairy industry, like cheese whey, were previously used for the production of recombinant proteins with expression levels similar to those obtained with IPTG (Viitanen et al. 2003; De León-Rodríguez et al. 2006).

All four newly developed copolymers were expressed at high levels using the optimised fermentation conditions. Treating the soluble crude lysate with an acidification treatment allowed removal of most of the *E*. *coli* endogenous proteins. This methodology was previously employed for the purification of a recombinant major ampullate spidroin I (MaSp1) protein from the spider *Nephila clavipes* (Xia et al. 2010). The crude lysate of MaSp1 producing cells was adjusted to pH 4 and led to precipitation of host cell proteins whereas the recombinant protein remained in the supernatant after centrifugation. However, the supernatant from the acidic treatment was still enriched with host cell proteins. In our study, we demonstrated that acidification at pH 3.0-3.5 is much more efficient for removal of *E*. *coli* endogenous proteins than pH 4. In fact, upon acidification of the SELP-1020-A crude lysate, a highly pure polymer fraction was obtained in just one step. Minor contaminants remained for SELP-520-A and SELP-59-A and these were removed by a simple ammonium sulphate precipitation step, followed by resuspension in cold water and dialysis against water.

SELPs are structural proteins lacking complex folding mechanisms and spontaneously self-assemble into β-sheets by hydrogen-bonding mediated processes. The stability of SELPs at low pHs is well known with many SELP copolymers being extensively processed into materials in formic acid solutions without any reported negative effects (Nagarajan et al. 2007; Qiu et al. 2009,2010). Furthermore, it was previously demonstrated that pH had no effect on the equilibrium swelling ratio of SELP hydrogels between pH 2.0 and pH 12.0, indicating an absence of pH sensitivity (Dinerman et al. 2002; Haider et al. 2005; Dandu et al. 2009). However, the substitution of an amino acid in the elastin-like block by a glutamic acid generated a pH stimuli-sensitive polymer (Nagarsekar et al. 2003, 2002). Here, we took advantage of this known acidic pH stability to separate the novel SELP polymers from the unstable contaminants. The acidic pH stability was further supported by comparing the FTIR spectra between pure lyophilized copolymer samples with and without the acidic treatment.

Concerning SELP-109-A, purification was not carried out as the polymer precipitated during cell lysis, possibly due to the high content of silk blocks that promote gelation (Megeed et al. 2002; Cappello et al. 1998). With the appropriate composition, SELPs undergo an irreversible sol–gel transition through crystallization of the silk-like blocks. This spontaneous transition is influenced by the number of silk-like blocks as well as by environmental conditions like temperature (Haider et al. 2004). As the formation of hydrogen-bonds is the major driving force behind gelation, copolymers with larger silk-like blocks will provide more contact points for potential inter-chain junctions (Haider et al. 2005; Cresce et al. 2008). On the other hand, the inclusion of larger elastin-like blocks will increase the spacing between the silk-like blocks, increasing its flexibility and aqueous solubility (Haider et al. 2005). In fact, when analysed by solution viscometry and under the same conditions, the rate of gelation was found to reflect the increase in silk-like block content (Cappello et al. 1998). In the case of SELP-109-A and due to the large silk-like and small interrupting elastin-like blocks, gelation occurs rapidly. A protocol for purification of SELP-109-A will be further developed, possibly involving the use of chaotropic agents in the lysis buffer in order to inhibit hydrogen bonding. However it is beyond the purpose of this work where our main focus is to report the genetic construction and especially, a facile method for recombinant SELP expression and purification. For the remaining copolymers, precipitation with ammonium sulphate revealed to be an easy method for completion of SELP purification, allowing for the obtaining of highly pure polymer fractions with relatively low concentrations of ammonium sulphate. The volumetric productivities after lyophilisation were 185, 151 and 198 mg/L for SELP-1020-A, SELP-520-A and SELP-59-A, respectively, in comparison to the previously described 25–30 mg/L purified with IMAC (Dandu et al. 2009; Haider et al. 2005; Nagarsekar et al. 2002).

In an attempt to improve and simplify the purification process further we investigated whether the pH 3.5 treatment may also allow for cell disruption and hence enable both protein release and purification in one-simple step. Our analysis indicated that the treatment was indeed just as efficient at cell disruption, as monitored by SDS-PAGE, as was sonication. Up to 0.8 g of cell pellets (wet weight) per ml of resuspension buffer, which was the maximum cell concentration that could be easily resuspended, were found to be efficiently disrupted by incubation at pH 3.5 with, in addition, the removal of the majority of contaminating proteins being also achieved. Indeed a comparative investigation of cell viability following treatment further supported the suitability of the approach for cell disruption as it was found to be even more effective in reducing cell viability than sonication. Further purification and protein concentration can be obtained by precipitation with 20% ammonium sulphate with subsequent resuspension and dialysis against water followed by lyophilisation allowing for the obtention of highly pure, stable polymers. Hence we have developed a simple non-chromatographic method that allows for both cell disruption and the obtention of highly pure protein with reduced cost. This facile method is suited for proteins expressed in the soluble form and stable at low pH as in the case of the structural proteins hereby exemplified.

SELP copolymers purified by the method developed in this study did not show any cytotoxicity in mouse myoblast cell line C2C12 and even presented a slight increase in cell viability [see Additional file 2]. This suggests that these novel SELPs can be processed into materials and explored for biomedical applications; which is currently under study at our group. Although this work is focused on lab-scale fermentations, the purification method has already been validated in a high-density fermentation experiment with a 500 L fermenter, showing full recovery of the recombinant copolymer with high purity.

## Competing interests

The authors declare that they have no competing interests.

## Supplementary Material

Additional file 1 Nucleotide and amino acid sequences of SELP constructions. Click here for file

Additional file 2 **Tetrazolium salt (MTS) test performed on C2C12 cells as a function of the concentration of SELP-1020-A and SELP-59-A.** Short term cell viability tests in response to SELP copolymer were performed by the MTS assay using the supplier’s recommended procedure (Promega). One millilitre of C2C12 cells at a concentration of 5 x 104 cells/mL were seeded in a 24-well culture plate and attached overnight in Dulbecco’s modified Eagle’s medium (DMEM) with 1% (v/v) fetal calf serum (FCS), 1% L-glutamine and no antibiotics at 37°C, 5% CO2, in a humidified environment. Lyophilized SELP was dissolved in PBS and then added to separate wells of the cell culture to achieve final concentrations of 2.5 μg/ml, 25 μg/ml and 250 μg/ml. The MTS assay was carried out after 5 days of culture. Standard culture media without copolymer were used as positive controls of cell viability. All the samples were tested in triplicate and the results expressed as percentage of the control (set as 100% viability). Click here for file
