# Supplementary material for: High level expression and facile purification of recombinant silk-elastin-like polymers in auto induction shake flask cultures
Source: AMB Express. 2013 Feb 5;3:11. doi: 10.1186/2191-0855-3-11 (PMC3599559; doi:10.1186/2191-0855-3-11)
Supplement: Additional file 1 — Nucleotide and amino acid sequences of SELP constructions. [file 2191-0855-3-11-S1.pdf]

High level expression and facile purification of recombinant silk-elastin-like polymers in auto induction shake flask cultures

Raul Machado, João Azevedo-Silva, Cristina Correia, Tony Collins, Francisco Javier Arias, Jose Carlos Rodríguez-Cabello, Margarida Casal

Additional file 1 – Nucleotide and amino acid sequences of SELP constructions.

SELP-109-A **$(S_{10}E_9)_7$**

Nucleotide Sequence

GTAGGTGCAGGCGCGGGTTCCGGCGCTGGTGCCGGCTCTGGTGCAGGCGCGGGTAGCGGCGCTGGTGCAGGCT
CCGGTGCGGGCGCTGGTTCTGGCGTAGGTGCAGGCGCGGGTTCCGGCGCTGGTGCCGGCTCTGGTGCAGGCGC
GGGTAGCGGCGCTGGTGCAGGCTCCGGTGCGGGCGCTGGTTCTGGCGTACCGGCCGTGGGCGTTCCAGCAGTG
GGTGTTCCGGCTGTGGGCGTTCCGGCGGTGGGTGTTCCGGCGGTGGGCGTTCCGGCTGTGGGTGTTCCGGCGG
TGGGCGTTCCTGCCGTGGGTGTTCCGGCAGTGGGCGTAGGTGCAGGCGCGGGTTCCGGCGCTGGTGCCGGCTC
TGGTGCAGGCGCGGGTAGCGGCGCTGGTGCAGGCTCCGGTGCGGGCGCTGGTTCTGGCGTAGGTGCAGGCGCG
GGTTCCGGCGCTGGTGCCGGCTCTGGTGCAGGCGCGGGTAGCGGCGCTGGTGCAGGCTCCGGTGCGGGCGCTG
GTTCTGGCGTACCGGCCGTGGGCGTTCCAGCAGTGGGTGTTCCGGCTGTGGGCGTTCCGGCGGTGGGTGTTCCG
GCGGTGGGCGTTCCGGCTGTGGGTGTTCCGGCGGTGGGCGTTCCTGCCGTGGGTGTTCCGGCAGTGGGCGTAG
GTGCAGGCGCGGGTTCCGGCGCTGGTGCCGGCTCTGGTGCAGGCGCGGGTAGCGGCGCTGGTGCAGGCTCCG
GTGCGGGCGCTGGTTCTGGCGTAGGTGCAGGCGCGGGTTCCGGCGCTGGTGCCGGCTCTGGTGCAGGCGCGGG
TAGCGGCGCTGGTGCAGGCTCCGGTGCGGGCGCTGGTTCTGGCGTACCGGCCGTGGGCGTTCCAGCAGTGGGT
GTTCCGGCTGTGGGCGTTCCGGCGGTGGGTGTTCCGGCGGTGGGCGTTCCGGCTGTGGGTGTTCCGGCGGTGG
GCGTTCCTGCCGTGGGTGTTCCGGCAGTGGGCGTAGGTGCAGGCGCGGGTTCCGGCGCTGGTGCCGGCTCTGG
TGCAGGCGCGGGTAGCGGCGCTGGTGCAGGCTCCGGTGCGGGCGCTGGTTCTGGCGTAGGTGCAGGCGCGGGT
TCCGGCGCTGGTGCCGGCTCTGGTGCAGGCGCGGGTAGCGGCGCTGGTGCAGGCTCCGGTGCGGGCGCTGGTT
CTGGCGTACCGGCCGTGGGCGTTCCAGCAGTGGGTGTTCCGGCTGTGGGCGTTCCGGCGGTGGGTGTTCCGGC
GGTGGGCGTTCCGGCTGTGGGTGTTCCGGCGGTGGGCGTTCCTGCCGTGGGTGTTCCGGCAGTGGGCGTAGGT
GCAGGCGCGGGTTCCGGCGCTGGTGCCGGCTCTGGTGCAGGCGCGGGTAGCGGCGCTGGTGCAGGCTCCGGT
GCGGGCGCTGGTTCTGGCGTAGGTGCAGGCGCGGGTTCCGGCGCTGGTGCCGGCTCTGGTGCAGGCGCGGGTA
GCGGCGCTGGTGCAGGCTCCGGTGCGGGCGCTGGTTCTGGCGTACCGGCCGTGGGCGTTCCAGCAGTGGGTGT
TCCGGCTGTGGGCGTTCCGGCGGTGGGTGTTCCGGCGGTGGGCGTTCCGGCTGTGGGTGTTCCGGCGGTGGGC
GTTCCTGCCGTGGGTGTTCCGGCAGTGGGCGTAGGTGCAGGCGCGGGTTCCGGCGCTGGTGCCGGCTCTGGTG
CAGGCGCGGGTAGCGGCGCTGGTGCAGGCTCCGGTGCGGGCGCTGGTTCTGGCGTAGGTGCAGGCGCGGGTTC
CGGCGCTGGTGCCGGCTCTGGTGCAGGCGCGGGTAGCGGCGCTGGTGCAGGCTCCGGTGCGGGCGCTGGTTCT
GGCGTACCGGCCGTGGGCGTTCCAGCAGTGGGTGTTCCGGCTGTGGGCGTTCCGGCGGTGGGTGTTCCGGCGG
TGGGCGTTCCGGCTGTGGGTGTTCCGGCGGTGGGCGTTCCTGCCGTGGGTGTTCCGGCAGTGGGCGTAGGTGCA
GGCGCGGGTTCCGGCGCTGGTGCCGGCTCTGGTGCAGGCGCGGGTAGCGGCGCTGGTGCAGGCTCCGGTGCG
GGCGCTGGTTCTGGCGTAGGTGCAGGCGCGGGTTCCGGCGCTGGTGCCGGCTCTGGTGCAGGCGCGGGTAGCG
GCGCTGGTGCAGGCTCCGGTGCGGGCGCTGGTTCTGGCGTACCGGCCGTGGGCGTTCCAGCAGTGGGTGTTCC
GGCTGTGGGCGTTCCGGCGGTGGGTGTTCCGGCGGTGGGCGTTCCGGCTGTGGGTGTTCCGGCGGTGGGCGTT
CCTGCCGTGGGTGTTCCGGCAGTGGGC

Amino Acid Sequence

V G A G A G S G A G A G S G A G A G S G A G A G S G A G A G S G V G A G A G S G A G A G S G A G A G S G A
G A G S G A G A G S G V P A V G V P A V G V P A V G V P A V G V P A V G V P A V G V P A V G V P A V G V P A
V G V G A G A G S G A G A G S G A G A G S G A G A G S G A G A G S G V G A G A G S G A G A G S G A G A G S
G A G A G S G A G A G S G V P A V G V P A V G V P A V G V P A V G V P A V G V P A V G V P A V G V P A V G V
P A V G V G A G A G S G A G A G S G A G A G S G A G A G S G A G A G S G V G A G A G S G A G A G S G A G A
G S G A G A G S G A G A G S G V P A V G V P A V G V P A V G V P A V G V P A V G V P A V G V P A V G V P A V
G V P A V G V G A G A G S G A G A G S G A G A G S G A G A G S G A G A G S G V G A G A G S G A G A G S G A
G A G S G A G A G S G A G A G S G V P A V G V P A V G V P A V G V P A V G V P A V G V P A V G V P A V G V P
A V G V P A V G V G A G A G S G A G A G S G A G A G S G A G A G S G A G A G S G V G A G A G S G A G A G S
G A G A G S G A G A G S G A G A G S G V P A V G V P A V G V P A V G V P A V G V P A V G V P A V G V P A V G
V P A V G V P A V G V G A G A G S G A G A G S G A G A G S G A G A G S G A G A G S G V G A G A G S G A G A
G S G A G A G S G A G A G S G A G A G S G V P A V G V P A V G V P A V G V P A V G V P A V G V P A V G V P A
V G V P A V G V P A V G V G A G A G S G A G A G S G A G A G S G A G A G S G A G A G S G V G A G A G S G A
G A G S G A G A G S G A G A G S G A G A G S G V P A V G V P A V G V P A V G V P A V G V P A V G V P A V G V
P A V G V P A V G V P A V G

SELP-59-A **$(S_5E_9)_9$**

Nucleotide Sequence

GTAGGTGCAGGCGCGGGTTCCGGCGCTGGTGCCGGCTCTGGTGCAGGCGCGGGTAGCGGCGCTGGTGCAGGCT
CCGGTGCGGGCGCTGGTTCTGGCGTACCGGCCGTGGGCGTTCCAGCAGTGGGTGTTCCGGCTGTGGGCGTTCC
GGCGGTGGGTGTTCCGGCGGTGGGCGTTCCGGCTGTGGGTGTTCCGGCGGTGGGCGTTCCTGCCGTGGGTGTT
CCGGCAGTGGGCGTAGGTGCAGGCGCGGGTTCCGGCGCTGGTGCCGGCTCTGGTGCAGGCGCGGGTAGCGGC
GCTGGTGCAGGCTCCGGTGCGGGCGCTGGTTCTGGCGTACCGGCCGTGGGCGTTCCAGCAGTGGGTGTTCCGG
CTGTGGGCGTTCCGGCGGTGGGTGTTCCGGCGGTGGGCGTTCCGGCTGTGGGTGTTCCGGCGGTGGGCGTTCC
TGCCGTGGGTGTTCCGGCAGTGGGCGTAGGTGCAGGCGCGGGTTCCGGCGCTGGTGCCGGCTCTGGTGCAGGC
GCGGGTAGCGGCGCTGGTGCAGGCTCCGGTGCGGGCGCTGGTTCTGGCGTACCGGCCGTGGGCGTTCCAGCAG
TGGGTGTTCCGGCTGTGGGCGTTCCGGCGGTGGGTGTTCCGGCGGTGGGCGTTCCGGCTGTGGGTGTTCCGGC
GGTGGGCGTTCCTGCCGTGGGTGTTCCGGCAGTGGGCGTAGGTGCAGGCGCGGGTTCCGGCGCTGGTGCCGGC
TCTGGTGCAGGCGCGGGTAGCGGCGCTGGTGCAGGCTCCGGTGCGGGCGCTGGTTCTGGCGTACCGGCCGTGG
GCGTTCCAGCAGTGGGTGTTCCGGCTGTGGGCGTTCCGGCGGTGGGTGTTCCGGCGGTGGGCGTTCCGGCTGT
GGGTGTTCCGGCGGTGGGCGTTCCTGCCGTGGGTGTTCCGGCAGTGGGCGTAGGTGCAGGCGCGGGTTCCGGC
GCTGGTGCCGGCTCTGGTGCAGGCGCGGGTAGCGGCGCTGGTGCAGGCTCCGGTGCGGGCGCTGGTTCTGGCG
TACCGGCCGTGGGCGTTCCAGCAGTGGGTGTTCCGGCTGTGGGCGTTCCGGCGGTGGGTGTTCCGGCGGTGGG
CGTTCCGGCTGTGGGTGTTCCGGCGGTGGGCGTTCCTGCCGTGGGTGTTCCGGCAGTGGGCGTAGGTGCAGGC
GCGGGTTCCGGCGCTGGTGCCGGCTCTGGTGCAGGCGCGGGTAGCGGCGCTGGTGCAGGCTCCGGTGCGGGC
GCTGGTTCTGGCGTACCGGCCGTGGGCGTTCCAGCAGTGGGTGTTCCGGCTGTGGGCGTTCCGGCGGTGGGTGT
TCCGGCGGTGGGCGTTCCGGCTGTGGGTGTTCCGGCGGTGGGCGTTCCTGCCGTGGGTGTTCCGGCAGTGGGC
GTAGGTGCAGGCGCGGGTTCCGGCGCTGGTGCCGGCTCTGGTGCAGGCGCGGGTAGCGGCGCTGGTGCAGGCT
CCGGTGCGGGCGCTGGTTCTGGCGTACCGGCCGTGGGCGTTCCAGCAGTGGGTGTTCCGGCTGTGGGCGTTCC
GGCGGTGGGTGTTCCGGCGGTGGGCGTTCCGGCTGTGGGTGTTCCGGCGGTGGGCGTTCCTGCCGTGGGTGTT
CCGGCAGTGGGCGTAGGTGCAGGCGCGGGTTCCGGCGCTGGTGCCGGCTCTGGTGCAGGCGCGGGTAGCGGC
GCTGGTGCAGGCTCCGGTGCGGGCGCTGGTTCTGGCGTACCGGCCGTGGGCGTTCCAGCAGTGGGTGTTCCGG
CTGTGGGCGTTCCGGCGGTGGGTGTTCCGGCGGTGGGCGTTCCGGCTGTGGGTGTTCCGGCGGTGGGCGTTCC
TGCCGTGGGTGTTCCGGCAGTGGGCGTAGGTGCAGGCGCGGGTTCCGGCGCTGGTGCCGGCTCTGGTGCAGGC
GCGGGTAGCGGCGCTGGTGCAGGCTCCGGTGCGGGCGCTGGTTCTGGCGTACCGGCCGTGGGCGTTCCAGCAG
TGGGTGTTCCGGCTGTGGGCGTTCCGGCGGTGGGTGTTCCGGCGGTGGGCGTTCCGGCTGTGGGTGTTCCGGC
GGTGGGCGTTCCTGCCGTGGGTGTTCCGGCAGTGGGC

Amino Acid Sequence

V G A G A G S G A G A G S G A G A G S G A G A G S G A G A G S G V P A V G V P A V G V P A V G V P A V G V P
A V G V P A V G V P A V G V P A V G V P A V G V G A G A G S G A G A G S G A G A G S G A G A G S G A G A G S
G V P A V G V P A V G V P A V G V P A V G V P A V G V P A V G V P A V G V P A V G V P A V G V P A V G V G A G A G S G
A G A G S G A G A G S G A G A G S G A G A G S G V P A V G V P A V G V P A V G V P A V G V P A V G V P A V G
V P A V G V P A V G V P A V G V G A G A G S G A G A G S G A G A G S G A G A G S G A G A G S G V P A V G V P
A V G V P A V G V P A V G V P A V G V P A V G V P A V G V P A V G V P A V G V P A V G V G A G A G S G A G A G S G A G
A G S G A G A G S G A G A G S G V P A V G V P A V G V P A V G V P A V G V P A V G V P A V G V P A V G V P A
V G V P A V G V G A G A G S G A G A G S G A G A G S G A G A G S G A G A G S G V P A V G V P A V G V P A V G
V P A V G V P A V G V P A V G V P A V G V P A V G V P A V G V G A G A G S G A G A G S G A G A G S G A G A G
S G A G A G S G V P A V G V P A V G V P A V G V P A V G V P A V G V P A V G V P A V G V P A V G V P A V G V
G A G A G S G A G A G S G A G A G S G A G A G S G A G A G S G V P A V G V P A V G V P A V G V P A V G V P A
V G V P A V G V P A V G V P A V G V P A V G V G A G A G S G A G A G S G A G A G S G A G A G S G A G A G S G
V P A V G V P A V G V P A V G V P A V G V P A V G V P A V G V P A V G V P A V G V P A V G

SELP-520-A **$(S_5E_{20})_5$**

Nucleotide Sequence

GTAGGTGCAGGCGCGGGTTCCGGCGCTGGTGCCGGCTCTGGTGCAGGCGCGGGTAGCGGCGCTGGTGCAGGCT
CCGGTGCGGGCGCTGGTTCTGGCGTACCGGCCGTGGGCGTTCCAGCAGTGGGTGTTCCGGCTGTGGGCGTTCC
GGCGGTGGGTGTTCCGGCGGTGGGCGTTCCGGCTGTGGGTGTTCCGGCGGTGGGCGTTCCGGCGGTGGGTGTT
CCGGCTGTGGGCGTTCCAGCCGTGGGTGTTCCGGCAGTGGGCGTACCGGCCGTGGGCGTTCCAGCAGTGGGTG
TTCCGGCTGTGGGCGTTCCGGCGGTGGGTGTTCCGGCGGTGGGCGTTCCGGCTGTGGGTGTTCCGGCGGTGGG
CGTTCCTGCCGTGGGTGTTCCGGCAGTGGGCGTAGGTGCAGGCGCGGGTTCCGGCGCTGGTGCCGGCTCTGGT
GCAGGCGCGGGTAGCGGCGCTGGTGCAGGCTCCGGTGCGGGCGCTGGTTCTGGCGTACCGGCCGTGGGCGTTC
CAGCAGTGGGTGTTCCGGCTGTGGGCGTTCCGGCGGTGGGTGTTCCGGCGGTGGGCGTTCCGGCTGTGGGTGTT
CCGGCGGTGGGCGTTCCGGCGGTGGGTGTTCCGGCTGTGGGCGTTCCAGCCGTGGGTGTTCCGGCAGTGGGCG
TACCGGCCGTGGGCGTTCCAGCAGTGGGTGTTCCGGCTGTGGGCGTTCCGGCGGTGGGTGTTCCGGCGGTGGG
CGTTCCGGCTGTGGGTGTTCCGGCGGTGGGCGTTCCTGCCGTGGGTGTTCCGGCAGTGGGCGTAGGTGCAGGC
GCGGGTTCCGGCGCTGGTGCCGGCTCTGGTGCAGGCGCGGGTAGCGGCGCTGGTGCAGGCTCCGGTGCGGGC
GCTGGTTCTGGCGTACCGGCCGTGGGCGTTCCAGCAGTGGGTGTTCCGGCTGTGGGCGTTCCGGCGGTGGGTGT
TCCGGCGGTGGGCGTTCCGGCTGTGGGTGTTCCGGCGGTGGGCGTTCCGGCGGTGGGTGTTCCGGCTGTGGGC
GTTCCAGCCGTGGGTGTTCCGGCAGTGGGCGTACCGGCCGTGGGCGTTCCAGCAGTGGGTGTTCCGGCTGTGGG
CGTTCCGGCGGTGGGTGTTCCGGCGGTGGGCGTTCCGGCTGTGGGTGTTCCGGCGGTGGGCGTTCCTGCCGTG
GGTGTTCCGGCAGTGGGCGTAGGTGCAGGCGCGGGTTCCGGCGCTGGTGCCGGCTCTGGTGCAGGCGCGGGTA
GCGGCGCTGGTGCAGGCTCCGGTGCGGGCGCTGGTTCTGGCGTACCGGCCGTGGGCGTTCCAGCAGTGGGTGT
TCCGGCTGTGGGCGTTCCGGCGGTGGGTGTTCCGGCGGTGGGCGTTCCGGCTGTGGGTGTTCCGGCGGTGGGC
GTTCCGGCGGTGGGTGTTCCGGCTGTGGGCGTTCCAGCCGTGGGTGTTCCGGCAGTGGGCGTACCGGCCGTGG
GCGTTCCAGCAGTGGGTGTTCCGGCTGTGGGCGTTCCGGCGGTGGGTGTTCCGGCGGTGGGCGTTCCGGCTGT
GGGTGTTCCGGCGGTGGGCGTTCCTGCCGTGGGTGTTCCGGCAGTGGGCGTAGGTGCAGGCGCGGGTTCCGGC
GCTGGTGCCGGCTCTGGTGCAGGCGCGGGTAGCGGCGCTGGTGCAGGCTCCGGTGCGGGCGCTGGTTCTGGCG
TACCGGCCGTGGGCGTTCCAGCAGTGGGTGTTCCGGCTGTGGGCGTTCCGGCGGTGGGTGTTCCGGCGGTGGG
CGTTCCGGCTGTGGGTGTTCCGGCGGTGGGCGTTCCGGCGGTGGGTGTTCCGGCTGTGGGCGTTCCAGCCGTG
GGTGTTCCGGCAGTGGGCGTACCGGCCGTGGGCGTTCCAGCAGTGGGTGTTCCGGCTGTGGGCGTTCCGGCGG
TGGGTGTTCCGGCGGTGGGCGTTCCGGCTGTGGGTGTTCCGGCGGTGGGCGTTCCTGCCGTGGGTGTTCCGGCA
GTGGGC

Amino Acid Sequence

V G A G A G S G A G A G S G A G A G S G A G A G S G A G A G S G V P A V G V P A V G V P A V G V P A V G V P
A V G V P A V G V P A V G V P A V G V P A V G V P A V G V P A V G V P A V G V P A V G V P A V G V P A V G V P
A V G V P A V G V P A V G V P A V G V P A V G V G A G A G S G A G A G S G A G A G S G A G A G S G A G A G S
G V P A V G V P A V G V P A V G V P A V G V P A V G V P A V G V P A V G V P A V G V P A V G V P A V G V P A V
G V P A V G V P A V G V P A V G V P A V G V P A V G V P A V G V P A V G V P A V G V P A V G V G A G A G S G
A G A G S G A G A G S G A G A G S G A G A G S G V P A V G V P A V G V P A V G V P A V G V P A V G V P A V G
V P A V G V P A V G V P A V G V P A V G V P A V G V P A V G V P A V G V P A V G V P A V G V P A V G V P A V G
V P A V G V P A V G V P A V G V G A G A G S G A G A G S G A G A G S G A G A G S G A G A G S G V P A V G V P
A V G V P A V G V P A V G V P A V G V P A V G V P A V G V P A V G V P A V G V P A V G V P A V G V P A V G V P
A V G V P A V G V P A V G V P A V G V P A V G V P A V G V P A V G V P A V G V G A G A G S G A G A G S G A G
A G S G A G A G S G A G A G S G V P A V G V P A V G V P A V G V P A V G V P A V G V P A V G V P A V G V P A
V G V P A V G V P A V G V P A V G V P A V G V P A V G V P A V G V P A V G V P A V G V P A V G V P A V G V P A
V G V P A V G

SELP-1020-A **$(S_{10}E_{20})_4$**

Nucleotide Sequence

GTAGGTGCAGGCGCGGGTTCCGGCGCTGGTGCCGGCTCTGGTGCAGGCGCGGGTAGCGGCGCTGGTGCAGGCT
CCGGTGCGGGCGCTGGTTCTGGCGTAGGTGCAGGCGCGGGTTCCGGCGCTGGTGCCGGCTCTGGTGCAGGCGC
GGGTAGCGGCGCTGGTGCAGGCTCCGGTGCGGGCGCTGGTTCTGGCGTACCGGCCGTGGGCGTTCCAGCAGTG
GGTGTTCCGGCTGTGGGCGTTCCGGCGGTGGGTGTTCCGGCGGTGGGCGTTCCGGCTGTGGGTGTTCCGGCGG
TGGGCGTTCCGGCGGTGGGTGTTCCGGCTGTGGGCGTTCCAGCCGTGGGTGTTCCGGCAGTGGGCGTACCGGC
CGTGGGCGTTCCAGCAGTGGGTGTTCCGGCTGTGGGCGTTCCGGCGGTGGGTGTTCCGGCGGTGGGCGTTCCG
GCTGTGGGTGTTCCGGCGGTGGGCGTTCCTGCCGTGGGTGTTCCGGCAGTGGGCGTAGGTGCAGGCGCGGGTT
CCGGCGCTGGTGCCGGCTCTGGTGCAGGCGCGGGTAGCGGCGCTGGTGCAGGCTCCGGTGCGGGCGCTGGTTC
TGGCGTAGGTGCAGGCGCGGGTTCCGGCGCTGGTGCCGGCTCTGGTGCAGGCGCGGGTAGCGGCGCTGGTGCA
GGCTCCGGTGCGGGCGCTGGTTCTGGCGTACCGGCCGTGGGCGTTCCAGCAGTGGGTGTTCCGGCTGTGGGCG
TTCCGGCGGTGGGTGTTCCGGCGGTGGGCGTTCCGGCTGTGGGTGTTCCGGCGGTGGGCGTTCCGGCGGTGGG
TGTTCCGGCTGTGGGCGTTCCAGCCGTGGGTGTTCCGGCAGTGGGCGTACCGGCCGTGGGCGTTCCAGCAGTGG
GTGTTCCGGCTGTGGGCGTTCCGGCGGTGGGTGTTCCGGCGGTGGGCGTTCCGGCTGTGGGTGTTCCGGCGGT
GGGCGTTCCTGCCGTGGGTGTTCCGGCAGTGGGCGTAGGTGCAGGCGCGGGTTCCGGCGCTGGTGCCGGCTCT
GGTGCAGGCGCGGGTAGCGGCGCTGGTGCAGGCTCCGGTGCGGGCGCTGGTTCTGGCGTAGGTGCAGGCGCG
GGTTCCGGCGCTGGTGCCGGCTCTGGTGCAGGCGCGGGTAGCGGCGCTGGTGCAGGCTCCGGTGCGGGCGCTG
GTTCTGGCGTACCGGCCGTGGGCGTTCCAGCAGTGGGTGTTCCGGCTGTGGGCGTTCCGGCGGTGGGTGTTCCG
GCGGTGGGCGTTCCGGCTGTGGGTGTTCCGGCGGTGGGCGTTCCGGCGGTGGGTGTTCCGGCTGTGGGCGTTC
CAGCCGTGGGTGTTCCGGCAGTGGGCGTACCGGCCGTGGGCGTTCCAGCAGTGGGTGTTCCGGCTGTGGGCGTT
CCGGCGGTGGGTGTTCCGGCGGTGGGCGTTCCGGCTGTGGGTGTTCCGGCGGTGGGCGTTCCTGCCGTGGGTG
TTCCGGCAGTGGGCGTAGGTGCAGGCGCGGGTTCCGGCGCTGGTGCCGGCTCTGGTGCAGGCGCGGGTAGCGG
CGCTGGTGCAGGCTCCGGTGCGGGCGCTGGTTCTGGCGTAGGTGCAGGCGCGGGTTCCGGCGCTGGTGCCGGC
TCTGGTGCAGGCGCGGGTAGCGGCGCTGGTGCAGGCTCCGGTGCGGGCGCTGGTTCTGGCGTACCGGCCGTGG
GCGTTCCAGCAGTGGGTGTTCCGGCTGTGGGCGTTCCGGCGGTGGGTGTTCCGGCGGTGGGCGTTCCGGCTGT
GGGTGTTCCGGCGGTGGGCGTTCCGGCGGTGGGTGTTCCGGCTGTGGGCGTTCCAGCCGTGGGTGTTCCGGCA
GTGGGCGTACCGGCCGTGGGCGTTCCAGCAGTGGGTGTTCCGGCTGTGGGCGTTCCGGCGGTGGGTGTTCCGG
CGGTGGGCGTTCCGGCTGTGGGTGTTCCGGCGGTGGGCGTTCCTGCCGTGGGTGTTCCGGCAGTGGGC

Amino Acid Sequence

V G A G A G S G A G A G S G A G A G S G A G A G S G A G A G S G V G A G A G S G A G A G S G A G A G S G A
G A G S G A G A G S G V P A V G V P A V G V P A V G V P A V G V P A V G V P A V G V P A V G V P A V G V P A
V G V P A V G V P A V G V P A V G V P A V G V P A V G V P A V G V P A V G V P A V G V P A V G V P A V G V P A
V G V G A G A G S G A G A G S G A G A G S G A G A G S G A G A G S G V G A G A G S G A G A G S G A G A G S
G A G A G S G A G A G S G V P A V G V P A V G V P A V G V P A V G V P A V G V P A V G V P A V G V P A V G V
P A V G V P A V G V P A V G V P A V G V P A V G V P A V G V P A V G V P A V G V P A V G V P A V G V P A V G V
P A V G V G A G A G S G A G A G S G A G A G S G A G A G S G A G A G S G V G A G A G S G A G A G S G A G A
G S G A G A G S G A G A G S G V P A V G V P A V G V P A V G V P A V G V P A V G V P A V G V P A V G V P A V
G V P A V G V P A V G V P A V G V P A V G V P A V G V P A V G V P A V G V P A V G V P A V G V P A V G V P A V
G V P A V G V G A G A G S G A G A G S G A G A G S G A G A G S G A G A G S G V G A G A G S G A G A G S G A
G A G S G A G A G S G A G A G S G V P A V G V P A V G V P A V G V P A V G V P A V G V P A V G V P A V G V P
A V G V P A V G V P A V G V P A V G V P A V G V P A V G V P A V G V P A V G V P A V G V P A V G V P A V G V P
A V G V P A V G
