# Supplementary material for: High level expression and facile purification of recombinant silk-elastin-like polymers in auto induction shake flask cultures
Source: AMB Express. 2013 Feb 5;3:11. doi: 10.1186/2191-0855-3-11 (PMC3599559; doi:10.1186/2191-0855-3-11)
Supplement: Additional file 2 — Tetrazolium salt (MTS) test performed on C2C12 cells as a function of the concentration of SELP-1020-A and SELP-59-A. Short term cell viability tests in response to SELP copolymer were performed by the MTS assay using the supplier’s recommended procedure (Promega). One millilitre of C2C12 cells at a concentration of 5 x 10^4^ cells/mL were seeded in a 24-well culture plate and attached overnight in Dulbecco’s modified Eagle’s medium (DMEM) with 1% (v/v) fetal calf serum (FCS), 1% L-glutamine and no antibiotics at 37°C, 5% CO2, in a humidified environment. Lyophilized SELP was dissolved in PBS and then added to separate wells of the cell culture to achieve final concentrations of 2.5 μg/ml, 25 μg/ml and 250 μg/ml. The MTS assay was carried out after 5 days of culture. Standard culture media without copolymer were used as positive controls of cell viability. All the samples were tested in triplicate and the results expressed as percentage of the control (set as 100% viability). [file 2191-0855-3-11-S2.pdf]

High level expression and facile purification of recombinant silk-elastin-like polymers in auto induction shake flask cultures

Raul Machado, João Azevedo-Silva, Cristina Correia, Tony Collins, Francisco Javier Arias, Jose Carlos Rodríguez-Cabello, Margarida Casal

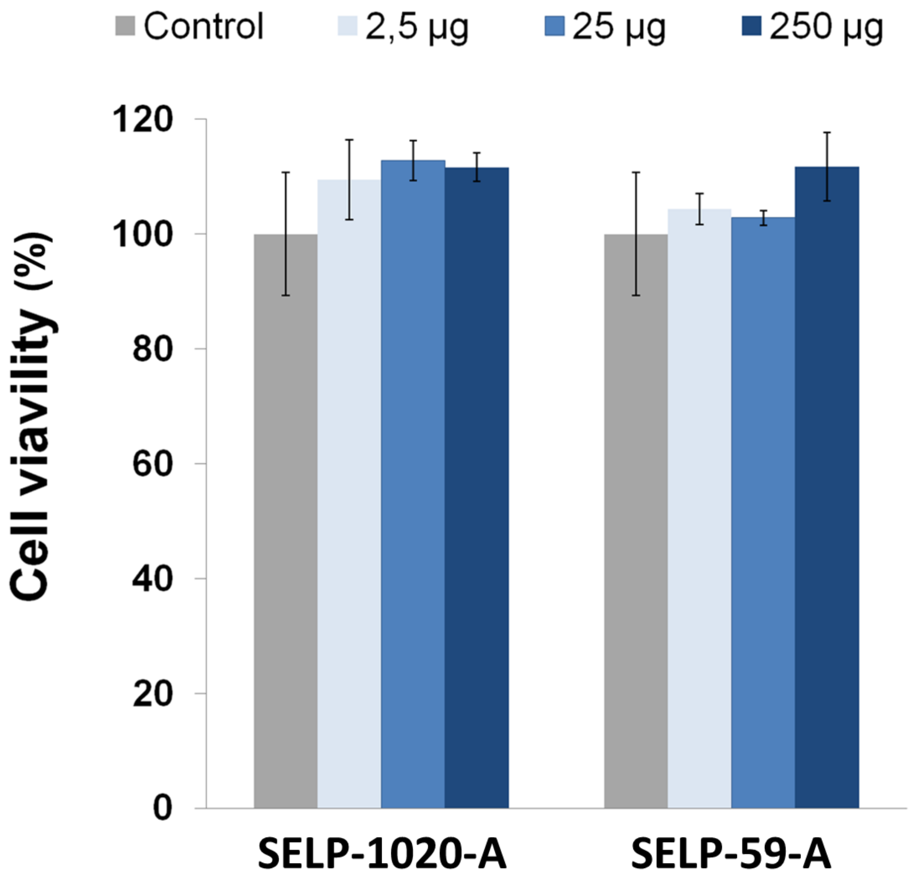

Additional File 2 – Tetrazolium salt (MTS) test performed on C2C12 cells as a function of the concentration of SELP-1020-A and SELP-59-A. Short term cell viability tests in response to SELP copolymer were performed by the MTS assay using the supplier's recommended procedure (Promega). One millilitre of C2C12 cells at a concentration of 5 x 104 cells/mL were seeded in a 24-well culture plate and attached overnight in Dulbecco's modified Eagle's medium (DMEM) with 1% (v/v) fetal calf serum (FCS), 1% L-glutamine and no antibiotics at 37 ºC, 5% CO2, in a humidified environment. Lyophilized SELP was dissolved in PBS and then added to separate wells of the cell culture to achieve final concentrations of 2.5 µg/ml, 25 µg/ml and 250 µg/ml. The MTS assay was carried out after 5 days of culture. Standard culture media without copolymer were used as positive controls of cell viability. All the samples were tested in triplicate and the results expressed as percentage of the control (set as 100% viability).
